# Effect of stacking sequence on the mechanical behaviour of CARALL and GLARE fiber metal laminates

**DOI:** 10.1038/s41598-026-50170-5

**Published:** 2026-04-24

**Authors:** Madhusudhan Balkundhi, Satish Shenoy Baloor, Gururaj Bolar

**Affiliations:** Manipal Institute of Technology, Manipal Academy of Higher Education, Manipal, India

**Keywords:** Fiber metal laminates, Composite manufacturing, Lightweight materials, Hybrid structural materials, Materials characterization, Engineering, Materials science

## Abstract

The present study systematically evaluates and compares the mechanical performance of CARALL_5 fiber metal laminates (FMLs) and GLARE_5 FMLs with two distinct stacking sequences. The mechanical performance was assessed considering the tensile strength, flexural strength, and impact strength of the two FMLs. Under tensile loading conditions, type-2 CARALL_5 FML exhibited a tensile strength of 776 ± 73 MPa, against a tensile strength of 466 ± 39 MPa exhibited by GLARE_5 FML. In comparison, type-1 CARALL FML, with aluminum as the outermost layers exhibited a comparatively lower tensile strength of 610 ± 53 MPa, while exhibiting a stepped failure pattern during tensile loading, indicating gradual progression of damage, and enhanced damage tolerance. The FMLs displayed failure modes such plastic deformation, fiber breakage, matrix cracking, and delamination under tensile loading conditions. The maximum values of flexural strength of around 1092 ± 85 MPa was observed in type-2 CARALL_5 FML, where the carbon fiber layers were on the outermost layers. In comparison, type-2 GLARE_5 FML displayed a 48% flexural strength, due to the lower elastic modulus and stiffness displayed by the glass fibers. Furthermore, type-1 CARALL_5 FML showed an impact energy of 14.2 ± 0.59 J, which was 98% greater than the impact energy of type-1 GLARE_5 FML. The observed failure modes were plastic deformation, fiber–matrix debonding, matrix cracking, and delamination. Based on these findings, type-1 CARALL_5 FMLs are ideal for applications involving tensile and impact loads, where it is crucial to retain load-bearing capacity even after sustaining damage. In contrast, type-2 CARALL_5 FML is more appropriate where the ability to endure bi-axial loading is essential.

**Supplementary Information:**

The online version contains supplementary material available at 10.1038/s41598-026-50170-5.

## Introduction

Fiber metal laminates (FMLs) are multi-layered composite materials formed by stacking alternate metal plies and fiber reinforced composites (FRCs), integrating the benefits of both metal and FRCs^1^. FMLs have gained immense popularity in recent years due to their adaptivity in material design and alignment with diverse loading conditions^2^. The mechanical properties of the FMLs can be curated by varying the materials of the metal ply, fiber material, orientation, and staking sequences^3^. Accordingly, FMLs are classified based on their constituent materials. Aluminum is the most used and widely accepted metallic material^4^. However, titanium, steel, and magnesium are also used as a substitute for aluminum. Important and widely used reinforced composite materials layers are manufactured using aramid, glass, or carbon fibers^5^. Accordingly, glass laminate aluminum reinforced epoxy (GLARE), carbon reinforced aluminum laminate (CARALL), aramid aluminum laminate (ARALL), and hybrid titanium composite laminate (HTCL) are the popular FMLs with wide industrial applications. FMLs display substantial improvement in the desirable properties compared to metals and composites^6^. Therefore, FMLs are widely used in aircraft industries. FMLs like GLARE and ARALL are used as skins in flaps and upper fuselage of an aircraft, lower wing structures, and as liners on the cargo bay floor^7^, while GLARE is used in Airbus A380 as a material for vertical stabilizer and lateral shell^8,9^. All the typical applications of the GLARE laminate with their stacking sequence are presented in Table 1^10^. At the same time, CARALL finds applications as impact absorbers in helicopters and as a desirable material for fabricating aircraft seats^8^.

**Table 1:** Stacking sequences available for GLARE with the application^10^.

GLARE type	Prepreg layup between the metal layer	Alloy and sheet thickness	Main beneficial characteristics	Typical application in an aircraft
GLARE 1	0° | 0°	AA7475-T761, 0.3–0.4 mm	Uni axial strength	Stiffeners
GLARE 2A	0° | 0°	AA-2024-T3, 0.2–0.5 mm	Uni axial strength	Stiffeners
GLARE 2B	90° | 90°		Strength perpendicular to the loading direction	Butt straps
GLARE 3	0° | 90°		Fatigue and impact strength	Bulkheads
GLARE 4A	0° | 90° | 0°		Fatigue and strength in 0°	Fuselage skins
GLARE 4B	90° | 0° | 90°		Fatigue and strength in 90°	Fuselage skins
GLARE 5	0° | 90° |90°| 0°		Impact strength	Floors, Cargo liners, Leading edges
GLARE 6A	+ 45° | − 45°		Shear strength	Blast-resistant luggage containers
GLARE 6B	+ 45° | − 45°			

FMLs offer a higher degree of practicability and have been consistently evaluated for their mechanical characteristics, considering different fiber orientations and stacking sequences application^11^. Taheri-Behrooz et al.^12^ explored the influence of four different metal-composite ply stacking sequences on the impact behavior of GLARE FML. Of the four FMLs tested, the FML with a stacking sequence of Al-glass-Al displayed higher load bearing capacity than the three other FMLs. The improvement in the load bearing capacity was attributed to the placement of aluminium as outer most layers and orientation of glass/epoxy laminates at 45°. Dhaliwal et al.^13^ analyzed the influence of stacking sequence on the dynamic response and failure mechanisms of CARALL FMLs under impact loading. The findings suggest that the symmetric laminates showcase slower crack propagation with improved energy absorption, whereas asymmetric laminates failed due to delamination and localized shear. Li et al.^14^ studied the flexural properties and failure mechanisms in GLARE FML under different orientations. Flexural strength and modulus increased with an increase in the number of fiber layers, attributing it to the increase in load-bearing members along the loading direction. However, in cross-ply laminates decrease in the bending modulus and strength was observed with the increase in the number of fiber layers. Azghan et al.^15^ found that the flexural properties of FMLs are significantly affected by the position of the high-strength fibers in the stacking sequence of FML. The flexural strength of basalt and glass fiber-based FMLs increased when the high-strength basalt fibers were placed at the outermost layers. Mohammed et al.^16^ compared the tensile strength of CARALL FML fabricated with different stacking sequence. Type-1 CARALL was developed by stacking the carbon fiber and aluminium alternatively, while type-2 CARALL was fabricated by sandwiching the carbon layers in between the two aluminium layers. It was noted that the tensile strength of the type-2 CARALL FML was 11% higher than type-1 FML. This increase in the strength was attributed to an increase in the fiber volume fraction. However, both types of CARALL FMLs displayed similar behavior under compressive loads. Bellini et al.^11^ studied the influence of aluminum and adhesive layers on the flexural strength of FML. The study revealed that the number of aluminum layers was the most influential factor for flexural strength with a contribution of 53.41%. However, the presence of an adhesive layer caused a degradation in the flexural strength. Arpatappeh et al.^17^ evaluated the effect of Kevlar and basalt fiber orientation on impact strength using Charpy impact test. The energy absorption of Kevlar fibers was higher in comparison with the basalt fiber layers and there was a progressive increase in the energy absorbed with an increase in number of basalt fiber layers. The analysis was also carried out on steel laminates with similar configurations. However, steel-based laminates suffered delamination because of weak epoxy adhesion. Hynes et al.^18^ experimentally evaluated stacking sequences in CARALL FMLs under tensile and flexural loading conditions. Based on the experimental work, the configuration Al/CFRP/Al/CFRP/Al exhibited higher tensile and flexural strength because of equitable load allocation to each layer. Hu et al.^19^ investigated the flexural behavior of CARALL FMLs through both experimental and numerical approaches. Their findings indicated that positioning the aluminum plies as exterior layers reduced stress concentrations at the aluminum-CFRP interface and enhanced the overall stiffness of FML. Furthermore, the study emphasized that stacking sequences play a crucial role in determining the onset of delamination and the failure modes during flexural loading. Hynes et al.^20^ conducted research on FMLs that incorporate both natural Jute fibers, and synthetic Kevlar fibers, arranged in various stacking orders. The combination of natural and synthetic fibers results in uniform load distribution, thereby increasing the stiffness of the FML. The Jute fibers played a crucial role in enhancing the mechanical properties due to their excellent bonding capabilities with both aluminum and Kevlar fibers. Gao et al.^21^ examined the behavior of CARALL FMLs with various stacking sequences subjected to flexural loading. It was noted that the bending modulus and strength increased with the fiber volume fraction, a result attributed to the robust carbon fibers. Nonetheless, the primary failure mechanisms in the FMLs were fiber breakage and matrix damage, which resulted in their complete failure. Fatima et al.^22^ investigated the influence of metal volume fraction in carbon, glass hybrid FMLs under tensile loading conditions. Increments in the metal volume fractions transformed the laminate behaviour from brittle to ductile nature. Subsequently, increasing the failure strain of the laminate. However, increasing the aluminum layers decreased the elastic modulus and tensile strength of the laminate. Kali et al.^23^ comparatively analyzed the impact behavior of GLARE FML with carbon and glass fiber-based hybrid FML with different stacking sequences. The hybridization of the FML enhanced the impact properties of the normal GLARE. This enhancement is attributed to the increased strength, stiffness, and effective bonding between carbon fibers and the polymer matrix. The laminates dissipate energy through mechanisms such as delamination, composite fracture, and fiber-matrix debonding. Ashraf et al.^24^ studied the low-velocity impact response of flax/basalt/aluminum hybrid composites in symmetric, asymmetric, and sandwich configurations. Accordingly, the asymmetric configuration exhibited higher impact resistance. The improvement is attributed to the specific arrangement of flax fibers, which enhanced the ductility. Wang et al.^25^ studied the tensile and flexural properties of a titanium-based FML with carbon and basalt fiber reinforcement under different stacking sequences. They discovered that fiber hybridization and the order of stacking influence the tensile and flexural failure modes. The outermost layers of the FML serve as the primary load-bearing member during tensile loading. Therefore, placing more basalt fibers in these outer layers can improve the flexural properties of the FML. Dhanaraj et al.^26^ explored the combined impact of pineapple and basalt fiber layers in improving tensile and flexural properties of the FML. The results demonstrated the basalt fibers contributed to increased durability, while pineapple fibers improved the flexibility of the hybrid FMLs. Subramanian et al.^27^ evaluated the response of titanium-based FMLs fabricated with novel woven (Jute and Kevlar) fiber mats under tensile, flexural, and impact loading conditions. The FMLs incorporating Kevlar fibers showed exceptional tensile and impact strengths, while those with Jute fibers had reduced strengths due to inadequate bonding. Nevertheless, the newly developed woven mats that combine Jute and Kevlar fibers managed to strike a right balance between offered strength and cost-effectiveness.

The existing literature has established the significance of stacking sequences and orientation in affecting the mechanical properties, such as tensile, flexural, and impact strength of FMLs. Selecting an appropriate stacking sequence is essential, as an unoptimized arrangement can result in uneven load distribution and stress concentration, potentially leading to early failure of the FML structures. Current research highlights the development and characterization of GLARE FMLs with suitable stacking configurations tailored for specific load-based applications (see Table 1). Nonetheless, the literature does not report studies concerning CARALL FMLs, suggesting an appropriate stacking sequence for a definitive load-bearing application, indicating a significant gap in the present state of research. The current gap in the research is attempted to be addressed by comparatively studying the effect of stacking sequences on the material behavior of CARALL FMLs with GLARE FMLs under varying loading conditions. Moreover, the distribution of load across individual layers under different loading conditions, particularly for CARALL, has not been thoroughly investigated. Understanding the failure mechanisms encountered by CARALL under different stacking sequences and loading conditions can provide deeper insights into the laminate’s behavior and applicability in real-world scenarios. Accordingly, CARALL_5 FMLs were developed with two distinct stacking sequences, and their mechanical properties were evaluated under tensile, flexural, and impact loading conditions. Furthermore, the mechanical properties of the developed CARALL_5 were compared with those of GLARE_5 FMLs. Additionally, the influence of different stacking sequences on mechanical properties and failure mechanisms was analyzed for both CARALL_5 and GLARE_5 FMLs.

## Materials and methods

The CARALL_5 and GLARE_5 laminates were prepared using aluminum 2024-T3 alloy having an ultimate tensile strength of 480 MPa and elastic modulus of 72.4 GPa. The alloy has a density of 2.78 g/cm^3^ and a Poisson’s ratio of 0.33. Carbon and glass fiber prepreg supplied by Bhor Chemicals and Plastics Pvt. Ltd. was used to fabricate CARALL_5 and GLARE_5, respectively. The prepreg properties were held constant for both carbon and glass fiber prepregs, with unidirectional orientation. The supplied prepregs contained A-45 epoxy resin with a resin content of 38 ± 3%, having a glass transition temperature of 125 °C. The fiber prepregs had an overall density of 200 GSM and a thickness of 0.2 mm. The composite density of carbon and glass fiber prepregs was 1.6 g/cm^3^.

**Fig. 1:**
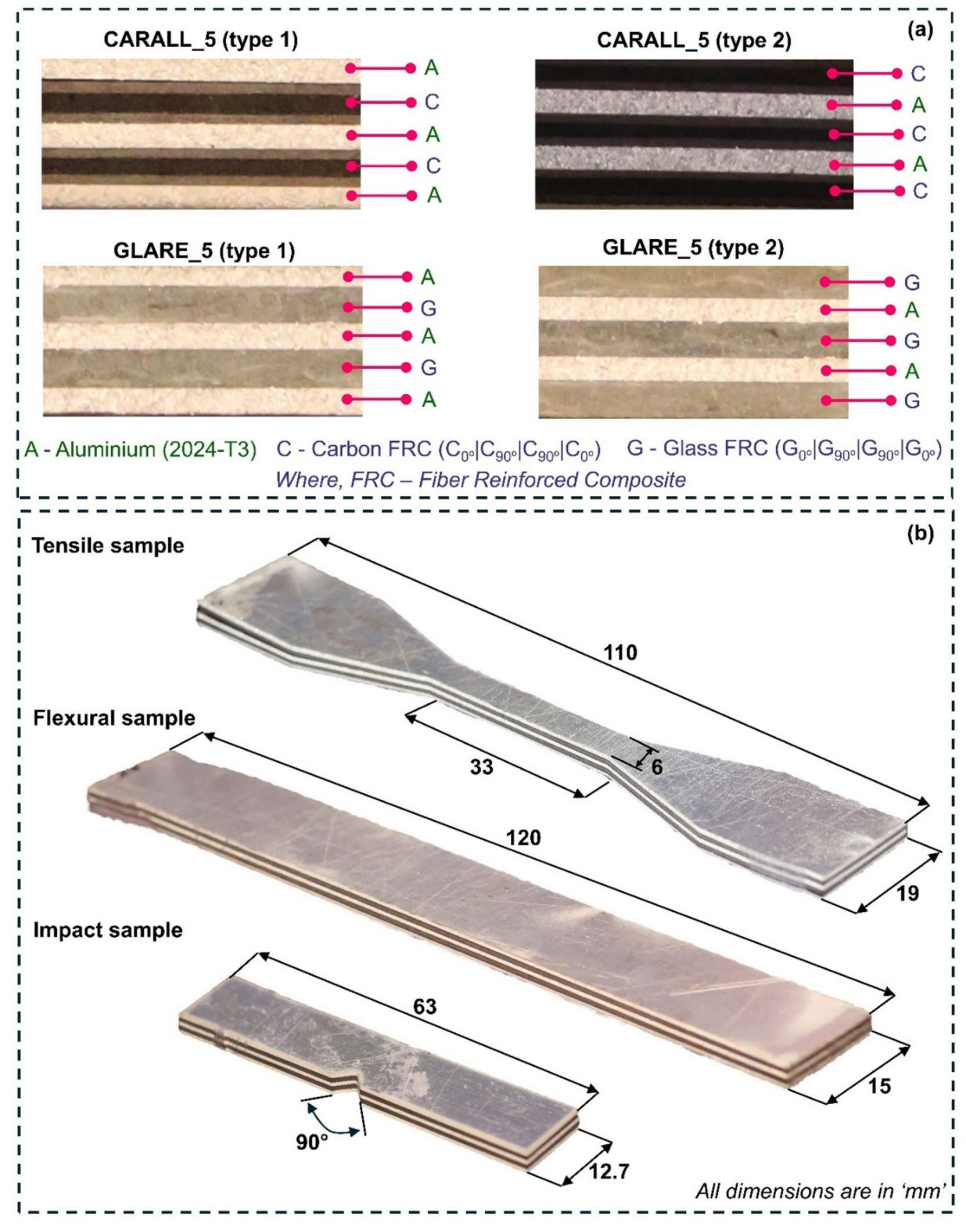
(**a**) Cross-sectional view of developed FMLs indicating the stacking sequence, (**b**) Pictorial view of specimens used for tensile, flexural, and impact testing.

The mechanical properties of CARALL_5 are analyzed in comparison to GLARE_5, using two distinct stacking sequences illustrated in Fig. 1a. The first configuration (type-1) follows the stacking order A|C|A|C|A, where ‘A’ denotes the aluminum layer and ‘C’ signifies four layers of carbon fiber prepreg arranged in the orientation C_0°_|C_90°_|C_90°_|C_0°_. Conversely, the second configuration (type-2) is arranged in the sequence C|A|C|A|C. Similarly, GLARE_5 FMLs were constructed using the same two stacking sequences to create type-1 GLARE_5 (A|G|A|G|A, where ‘G’ stands for four layers of glass fiber prepreg oriented as G_0°_|G_90°_|G_90°_|G_0°_) and type-2 GLARE_5 (G|A|G|A|G). The average thickness and metal volume fractions of these laminates are detailed in Table 2.

**Table 2:** Average thickness and metal volume fraction of FMLs.

Specimen	Average thickness (mm)	Number of metal layers	Metal volume fraction
CARALL_5 (type 1)	3.21	3	0.46
CARALL_5 (type 2)	3.31	2	0.28
GLARE_5 (type 1)	3.20	3	0.46
GLARE_5 (type 2)	3.32	2	0.27

**Fig. 2:**
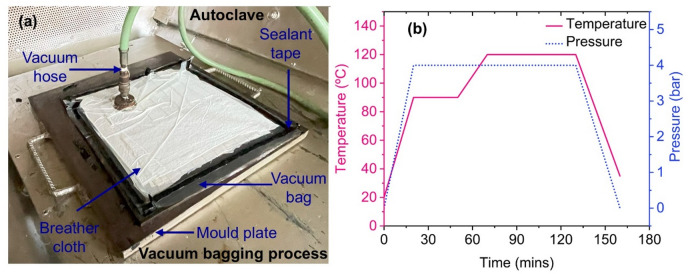
(**a**) Vacuum bagging method to cure FMLs in the autoclave (**b**) Curing cycle employed.

The aluminum layer underwent surface treatment to ensure effective bonding with the composite layers. Initially, the aluminum surface was cleaned with a NaOH solution for 10 s to eliminate oil and grease. Later the surface was dried using hot air and subjected to anodizing treatment. The anodizing process involved using 0.5 M sulfuric acid for 20 min at room temperature, which resulted in the formation of nanosized pores on the surface, enhancing the wettability and adhesion strength of the aluminum alloy^28^. The treated aluminum surface and the necessary fiber prepregs were arranged according to the sequence shown in Fig. 1a. The specimens were cured in an autoclave using the vacuum bagging technique as depicted in Fig. 2a. The curing cycle is detailed in Fig. 2b. Initially, the laminates were cured at 90 °C for 30 min, then the temperature increased to 120 °C at a rate of 2 °C/min. The final curing stage was conducted at 120 °C for 60 min, followed by cooling to room temperature at a rate of 3 °C/min. The entire curing process was maintained at a constant pressure of 4 bar. The required test specimens were cut using Abrasive Water Jet Machining (AWJM) from the prepared laminates according to the dimensions specified in Fig. 1b.

**Fig. 3:**
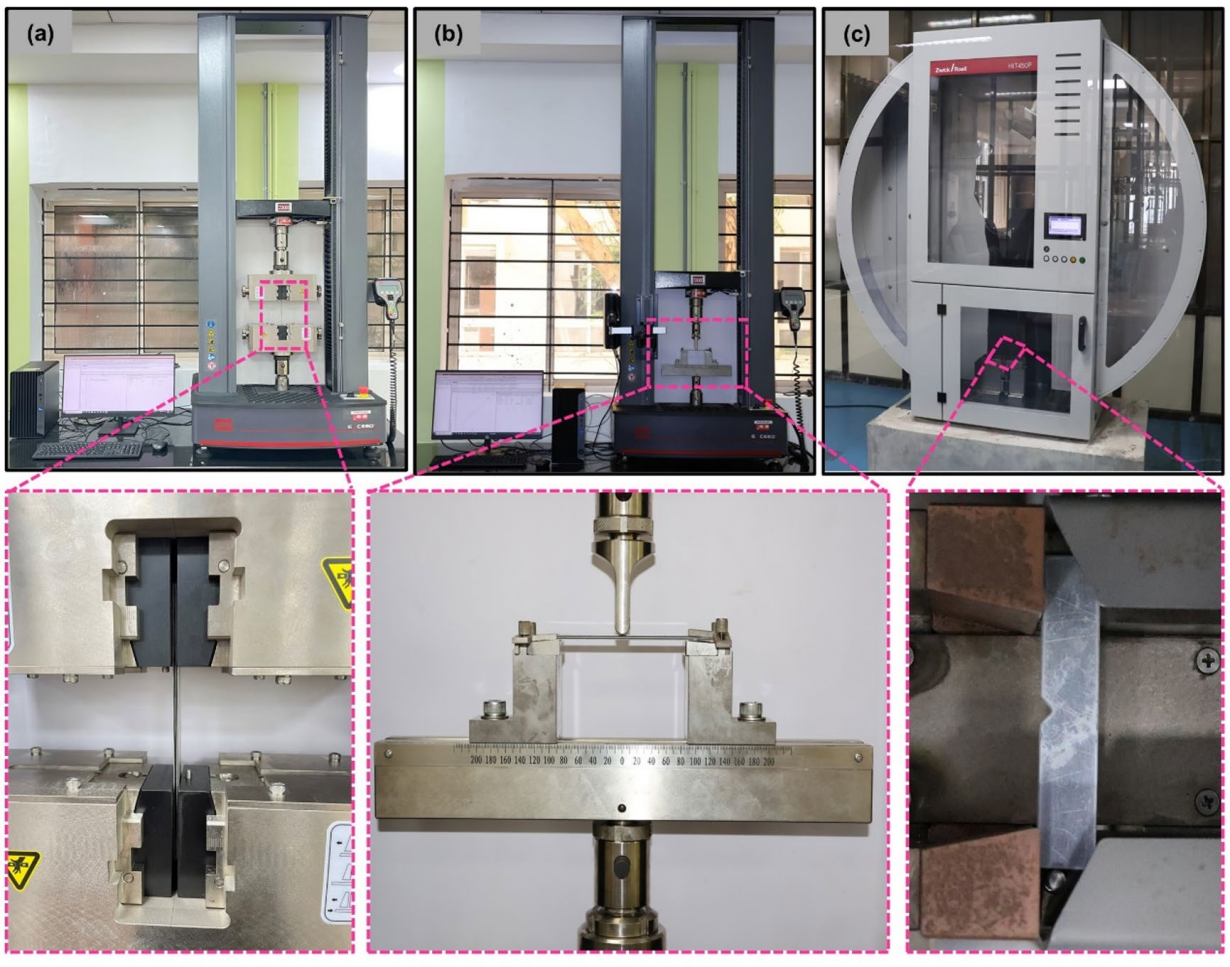
Pictorial view of experimental setups for (**a**) Tensile test (**b**) Flexural test (**c**) Impact test.

The fabricated CARALL_5 and GLARE_5 laminates were subjected to tensile, flexural, and impact-loading tests. The tensile and flexural assessments were conducted following ASTM D638 and ASTM D790 standards, respectively. The Charpy impact test adhered to the ASTM D256 standard. The tensile and flexural tests were performed at a steady rate of 2 mm/min using the MTS-Exceed series universal testing machine with a 50 kN capacity. Impact testing was executed with a Zwick-Roell instrumented impact tester, which has a maximum capacity of 50 J and a rising angle of 150°. Specimens were prepared with a 90° V-notch for the edgewise impact analysis. Figure 3a–c illustrates the experimental setups for tensile, flexural, and impact testing of the samples. Each test was repeated five times, and the average results are reported.

## Results and discussion

### Tensile strength analysis

**Fig. 4:**
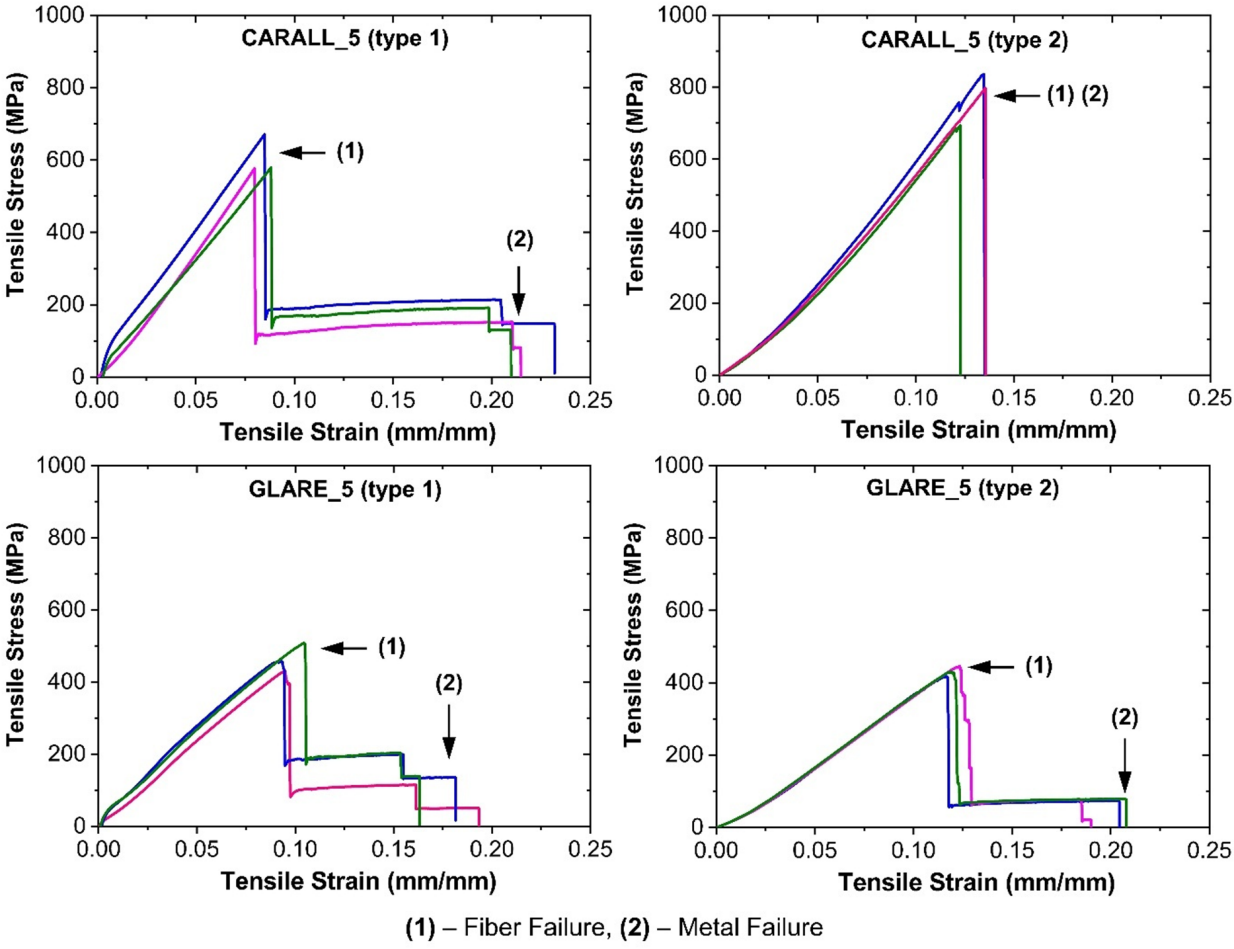
Tensile stress–strain curves of FMLs under tensile loading.

The tensile stress–strain behavior of the developed CARALL_5 and the GLARE_5 FMLs is presented in Fig. 4. Upon examining the stress–strain curve, it becomes evident that CARALL_5 FML can endure greater stress levels than GLARE_5 FML. In scenarios involving uniaxial loading, much of the load is supported by fibers oriented at 0°, where the strength of the fibers in the direction of the fiber alignment is of utmost importance. The primary reason CARALL_5 FMLs have higher failure thresholds than GLARE_5 FMLs is linked to the remarkable properties exhibited by carbon fibers compared to the glass fibers. Carbon fibers possess significantly higher tensile strength (4000 MPa) and elastic modulus (240 GPa) compared to glass fibers, having a tensile strength of 3750 MPa and modulus of 80 GPa. This allows CARALL laminates to endure greater applied stresses and facilitates more efficient load transfer from the aluminum layers to the fiber reinforcement. This improved load-bearing capacity delays the initiation of critical damage mechanisms such as fiber fracture, matrix cracking, and interfacial delamination. Conversely, the relatively lower stiffness, strength and modulus of glass fibers in GLARE_5 restricts their capacity to endure high tensile stresses.

Furthermore, when the stress–strain behavior of type-1 and type-2 CARALL FMLs are compared, the failure of fiber-reinforced composite and metal layers occurred instantaneously in the type-2 CARALL FML. Whereas, in the case of type-1 CARALL_5 FML, a clear distinction between composite and metal layer failure was observed. The type-1 CARALL_5 FML, which has a higher metal volume fraction of 46%, gains from improved load distribution and energy absorption due to its aluminum layers. The type-1 CARALL_5 FML attained an ultimate tensile stress value nearing 600 MPa. Until the point of ultimate stress, the load was supported by both metal and fiber layers. After the fiber composite fails at point (1) as seen in Fig. 4, the load is borne by the aluminum layers of the FML. However, the higher metallic content in the FML enables the aluminum to plastically deform even after the fracture initiation in fiber composites, thus postponing catastrophic failure of FML. This leads to a step-by-step failure process, where fiber composite failure occurs before metal failure, clearly distinguishing the two failure events. Conversely, the immediate failure of both the fiber composite and metal layers in the type-2 CARALL FML is mainly due to its lower metal volume fraction of 28%, which restricts the aluminum layers ability to share the load and undergo plastic deformation. Due to the higher fiber volume fraction, type-2 CARALL_5 FMLs were able to attain an ultimate tensile stress of around 780 MPa. The intermediate aluminum layers also assisted in carrying the load until the point of ultimate stress. However, the failure of fiber composite layers at the ultimate stress point transferred the entire load to the intermediate aluminum layers. When the aluminum sheets were subjected to a sudden load far exceeding their ultimate tensile strength, the strain-hardened aluminum experienced a catastrophic failure. Consequently, the stress–strain curves showed no distinct points of failure between the metal and the composite fiber layers.

Conversely, stress–strain curves for GLARE_5 illustrated in Fig. 4 suggest that the stacking sequence has a relatively minor effect on ultimate stress and failure behavior of GLARE FMLs.

The comparatively lower influence of stacking sequence on the tensile stress–strain behavior of GLARE_5 FMLs is due to the similar tensile strengths of the materials involved, specifically aluminum 2024-T3 and glass fiber–reinforced polymer layers. Because the ultimate tensile strength of glass/epoxy composite layers (360 MPa) is close to that of aluminum (around 480 MPa), the load applied is more evenly distributed between the metal and fiber layers, regardless of their stacking arrangement. This leads to comparable deformation and failure patterns across different stacking sequences, resulting in a slight difference in the stress–strain curves of type-1 and type-2 GLARE_5 FMLs. However, CARALL FMLs are composed of layers of aluminum and carbon fiber/epoxy composite, which exhibit a significant difference in tensile strength, with carbon/epoxy composite layers possessing a much higher tensile strength of approximately 774 MPa. This considerable disparity makes the load transfer and failure progression highly dependent on the stacking arrangement, rendering the stacking sequence a crucial element in determining the mechanical behavior of CARALL FMLs.

**Fig. 5:**
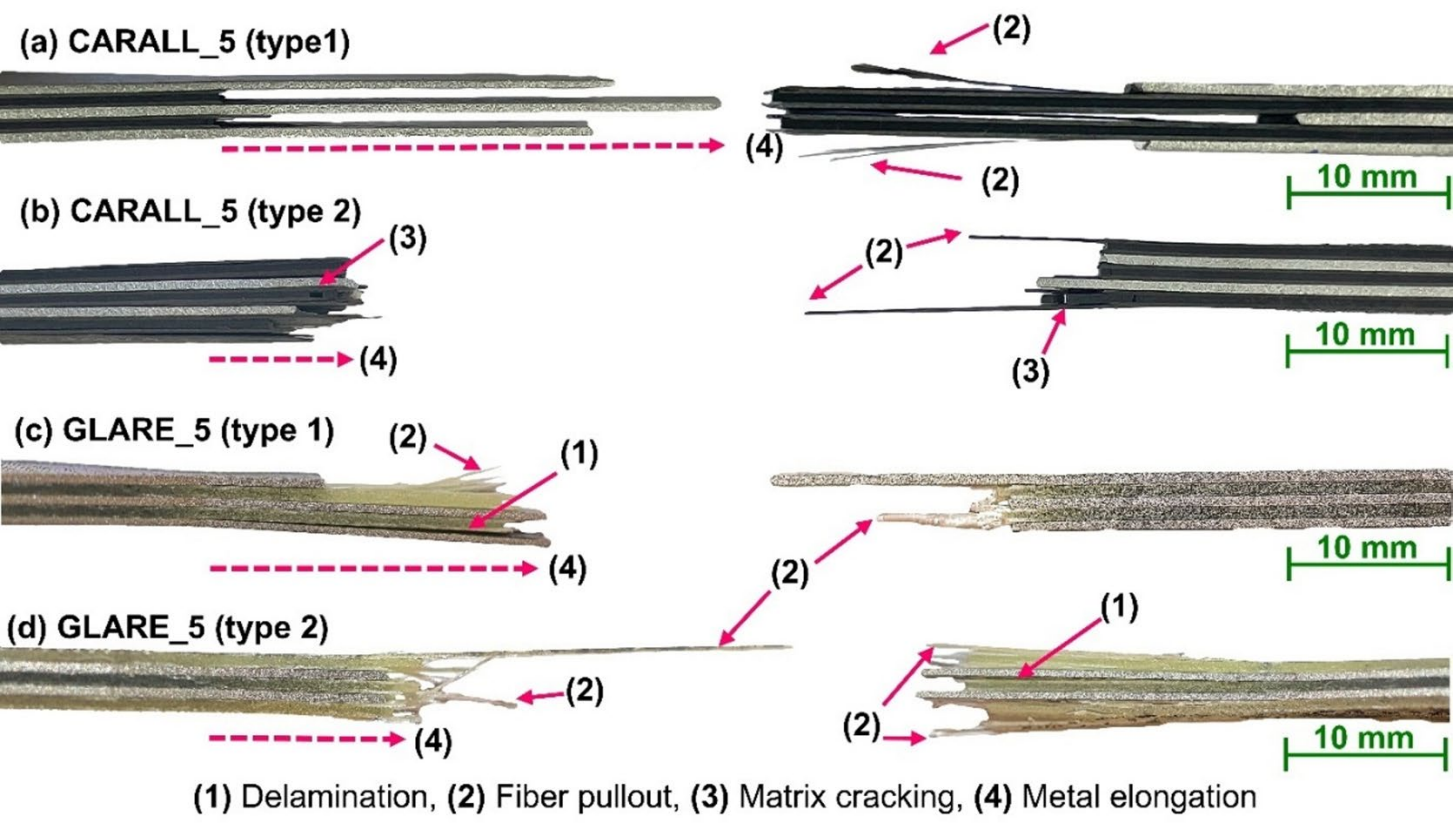
Side profiles of failed FMLs under tensile loading.

**Fig. 6:**
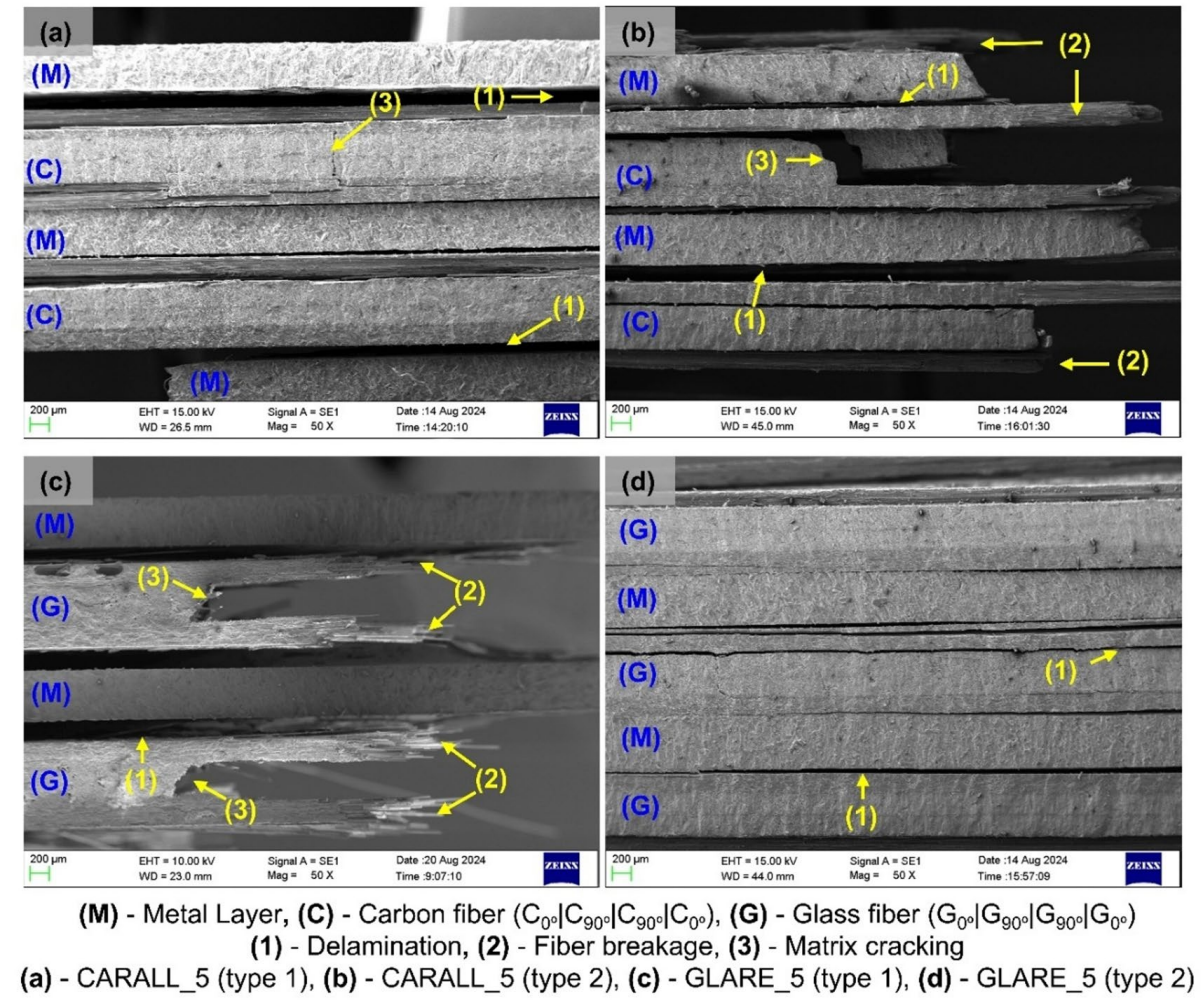
SEM images of a failed FMLs under tensile loading.

Additionally, an examination of the failed specimens was conducted. Figure 5 illustrates the side view of the specimens that failed under tensile loading, while Fig. 6 presents the micrographs of the failed specimens obtained via scanning electron microscopy. Type-1 CARALL_5 FML exhibited significant elongation of aluminum layers before failure compared to type-2 CARALL_5 FML (see Fig. 5a and b). As seen in Fig. 4, type-1 CARALL_5 FML experienced fiber-reinforced composite and metal layer failure at different stages. Even after the fiber composite failure at point (1), the aluminum layers continued to support the load, undergoing significant stretching before failure. Therefore, the SEM micrographs of type-1 CARALL_5 showcased a less severe failure mode, such as delamination and matrix cracking, as seen in Fig. 6a. In contrast, type-2 CARALL_5 FML saw simultaneous failure of metal and fiber-reinforced composite layers, which limited the deformation and elongation of aluminum layers. The type-2 CARALL FML displayed severe delamination, fiber pull-out, and matrix cracking, as depicted in Fig. 6b. Furthermore, the failure pattern of GLARE_5 FML was akin to that of CARALL_5 FMLs, with type-1 GLARE_5 FML showing aluminum layer elongation compared to type-2 GLARE_5 FML (see Fig. 5c and d). This is again attributed to the higher metal volume fraction in type-1 GLARE_5 FML. SEM analysis of the GLARE_5 FMLs indicated that delamination was the dominant failure mode in both type-1 and type-2 stacking sequences, as shown in Fig. 6c and d. Additionally, in all the four FMLs examined, fibers aligned at 0° experienced failure due to fiber breakage, while those aligned at 90° failed because of matrix cracking.

**Fig. 7:**
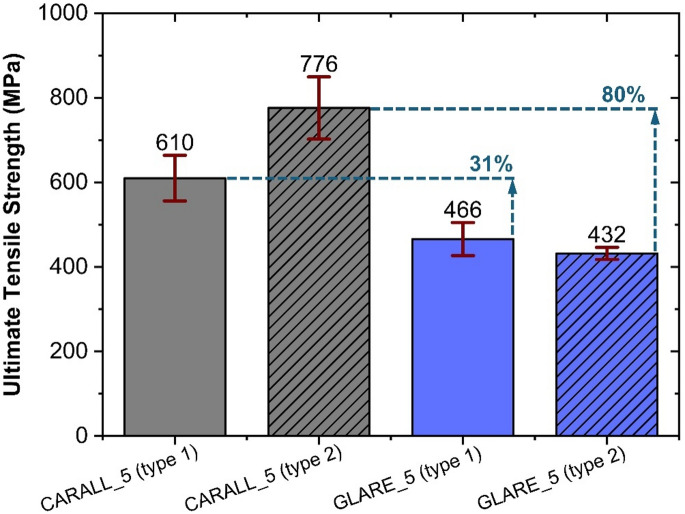
Ultimate tensile strength of the developed FMLs.

Figure 7 presents the ultimate tensile strength of the type-1 and type-2 CARALL and GLARE FMLs. CARALL_5 FML exhibits greater tensile strength than GLARE_5 FML across both stacking sequences. The type-1 CARALL_5 FML achieved an ultimate tensile strength of 610 ± 53 MPa, surpassing the ultimate tensile strength of type-1 GLARE_5 FML (466 ± 39 MPa) by 31%. The type-2 CARALL_5 FML displayed an ultimate tensile strength of 776 ± 73 MPa, which is 80% higher than the ultimate tensile strength of type-2 GLARE_5 FML (432 ± 14 MPa). The enhanced tensile strength of CARALL_5 FMLs is attributed to the higher strength and good bonding ability of carbon fibers. Additionally, the stepped failure pattern observed in type-1 stacking sequence of CARALL indicates a gradual progression of damage, effective load redistribution, and enhanced damage tolerance. Such a response provides early warning prior to ultimate failure, allowing inspection or maintenance to be performed before catastrophic failure. This makes type-1 CARALL FML well-suited for applications where maintaining residual load-carrying capacity after damage is critical.

**Fig. 8:**
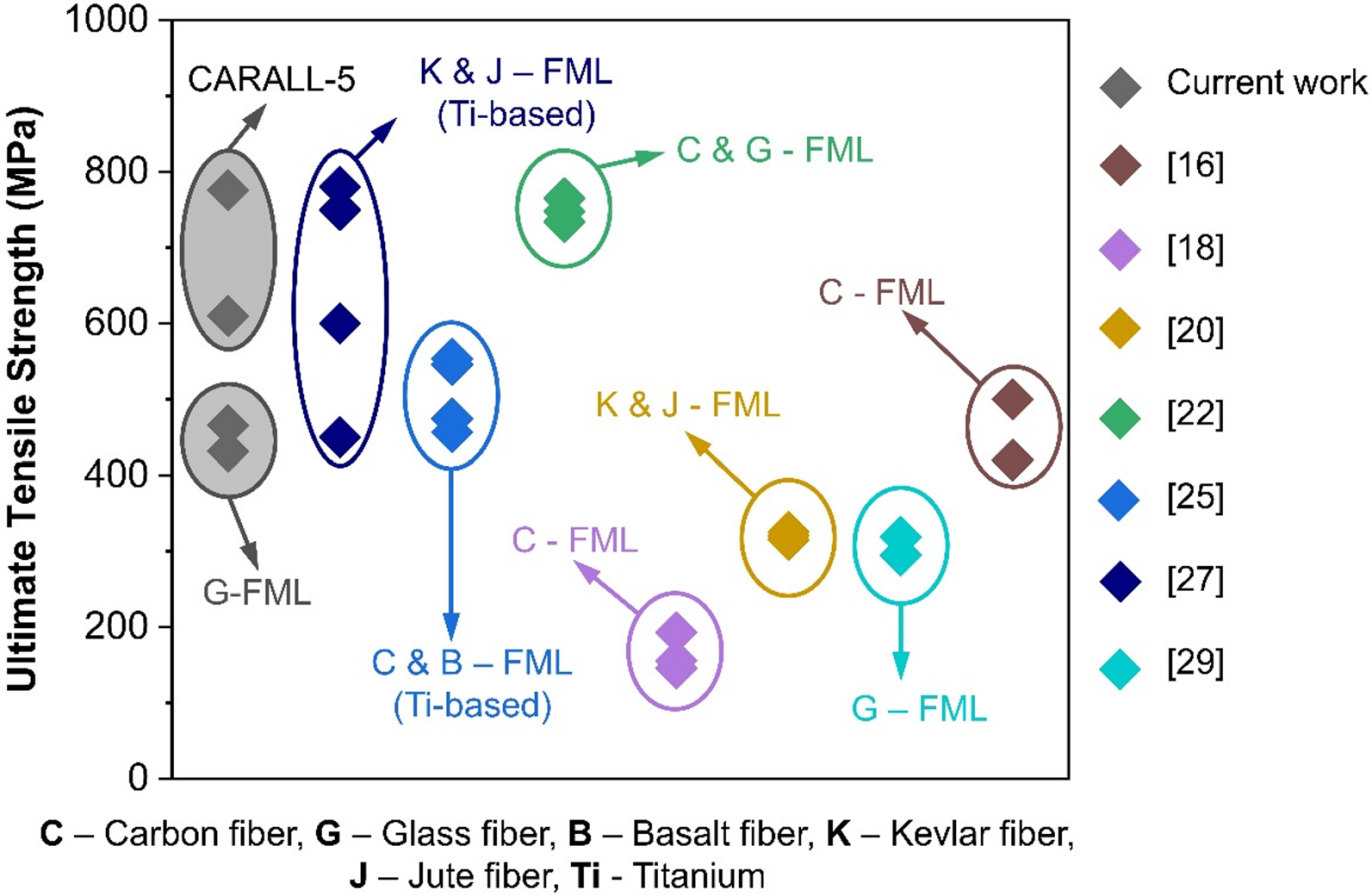
Comparative analysis for tensile strength of CARALL_5 with previous studies.

Furthermore, the measured ultimate tensile strength for developed CARALL_5 was compared with the previous studies^16,18,20,22,25,27,29^, as shown in Fig. 8. For comparison, aluminum-based FMLs reported in the literature were considered, along with recent reports on titanium-based FMLs. Additionally, the detailed summary of the literature is provided in Table S1 of the supplementary information. The comparative analysis highlights the potential utility of CARALL_5 over other FMLs documented in the literature. The tensile strength values for the developed CARALL_5 FMLs were found to be superior to most reported FMLs. However, the titanium-based FML developed by Subramanian et al.^27^ demonstrated similar performance to the CARALL_5 FML. The enhanced tensile strength of the titanium sheets was identified as a key factor in boosting the tensile strength of titanium-based FMLs. Additionally, the tensile strength of carbon and glass fiber hybrid FMLs ranged from 765 to 734 MPa^22^, which aligns with the findings of the current study where tensile strength as high as 776 MPa was measured for type-2 CARALL FML. Consequently, the developed CARALL_5 FMLs are suitable for applications where damage tolerance and resistance to uniaxial tensile loading are critical requirements.

### Flexural strength analysis

**Fig. 9:**
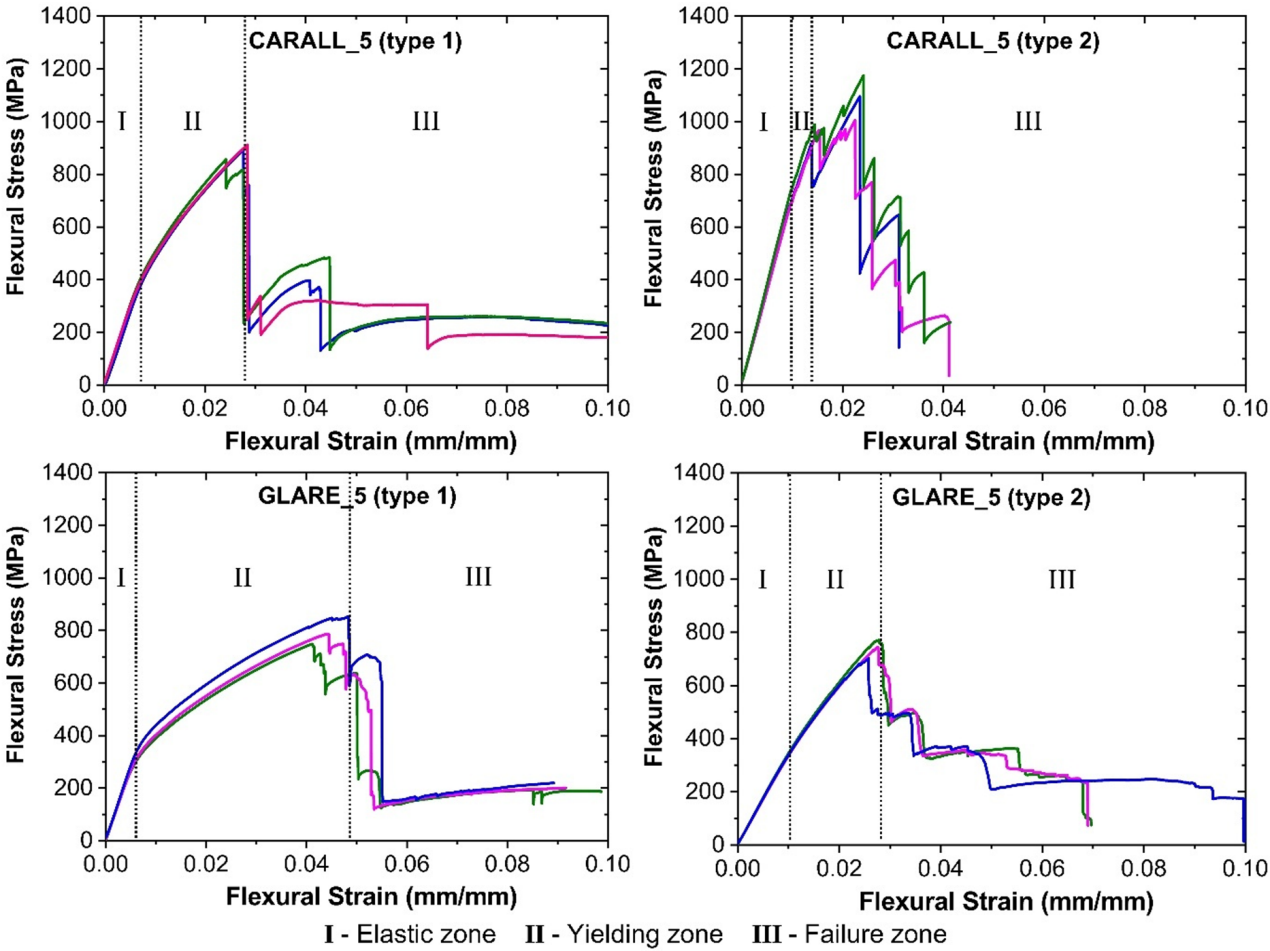
Flexural stress–strain curves under flexural loading.

Figure 9 illustrates the flexural stress–strain curves for both type-1 and type-2 CARALL_5 and GLARE_5 FMLs. The flexural behavior of CARALL_5 and GLARE_5 can be examined through three distinct phases. In stage 1, the FMLs demonstrate elastic properties, with both aluminum and fiber composite layers undergoing elastic deformation. During this first phase, stress and strain maintain an almost linear correlation, made possible by the robust interfacial bonding between the aluminum and fiber layers. Additionally, no damage is detected in the material at this stage, indicating that the FML can be safely loaded up to this point without sustaining any significant damage.

**Fig. 10:**
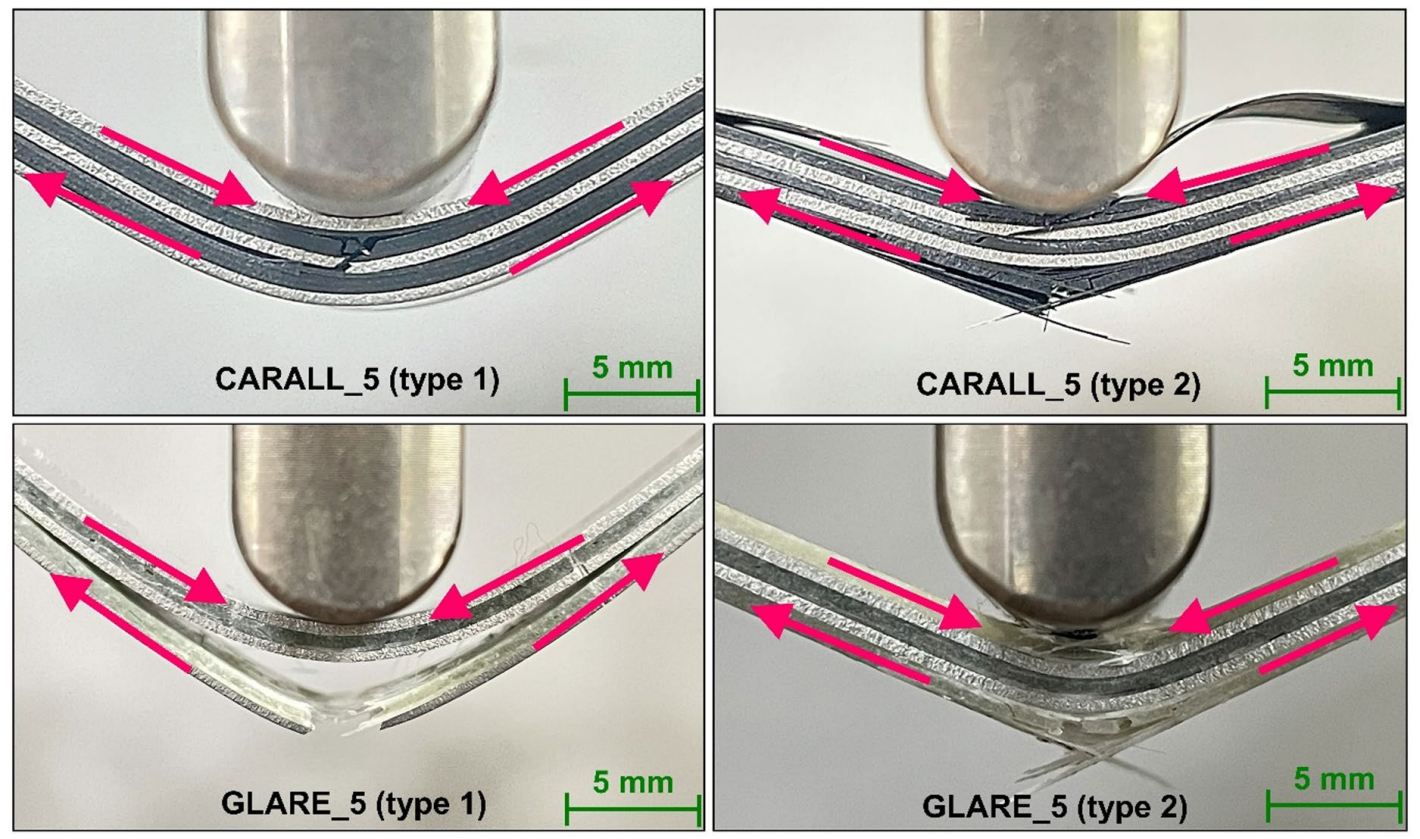
Side-profiles of CARALL_5 and GLARE_5 FMLs during flexural loading.

As the crosshead displacement continues to increase, the aluminum begins to yield while the fibers remain intact in the case of type-1 CARALL and GLARE FMLs. This is illustrated by a change in the trajectory and slope of the curves in stage 2. In this stage, the stress–strain curve for the type-1 CARALL and GLARE FMLs exhibits a change in slope with pronounced strain, indicating yielding of the metal layers and greater elastic deflection under the same applied load. Whereas in the case of type-2 CARALL and GLARE FMLs, very little change in the trajectory and slope of the curves was observed, as depicted in Fig. 9. This can be attributed to the higher volume fraction and high stiffness of the fibers, which limit elastic deformation. The degree of slope change influences the width of the second stage, which can be observed from Fig. 9. Type 1 FMLs due to increased yielding resulted in a longer second stage in comparison with type 2 FMLs, with a shorter yielding period. Moreover, the fiber composite layers continue to bear most of the load, thus preventing the metal layers from yielding. Moreover, GLARE FMLs resulted in a higher width of the second stage than CARALL FMLs, attributing to the improved flexibility offered by the glass fibers over carbon fibers. By the end of stage 2, the FMLs are subjected to loads exceeding their capacity, leading to the failure of the constituent materials, as illustrated in Fig. 10. In stage 3, all four FMLs experienced a reduction in flexural strength at various points of crosshead displacement. These significant strength drops are mainly attributed to fiber layer breakage, interface failure, and metal fracture.

Additional insights from Fig. 9 indicate that type-2 CARALL_5 FML can endure greater flexural stress compared to type-1 CARALL FML. This enhanced load-bearing capability of the type-2 FML is due to the optimal positioning of high-strength carbon fibers. When subjected to flexural loading, materials experience significant compressive stress on the top layer and tensile stress on the bottom layer. In Type-1 CARALL FML, aluminum forms the topmost and bottommost layers, handling both tensile and compressive loads. Conversely, in the type-2 CARALL FML, carbon fibers are positioned at the topmost and bottommost layers, primarily bearing the load. The placement of high-strength carbon fibers at points of maximum tensile and compressive stresses allows the type-2 FML to support higher flexural loads.

**Fig. 11:**
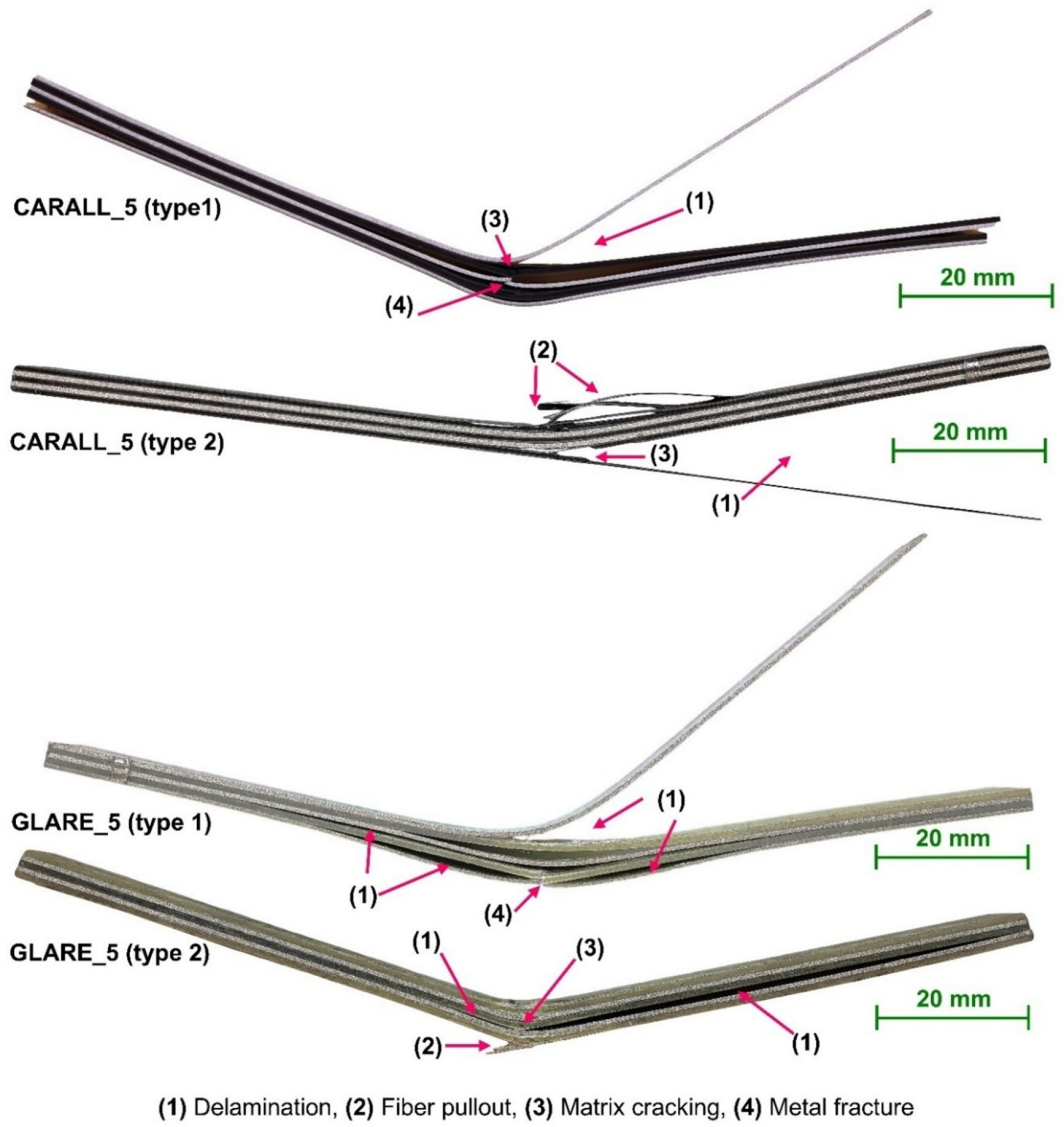
Side profiles of failed FMLs under flexural loading.

**Fig. 12:**
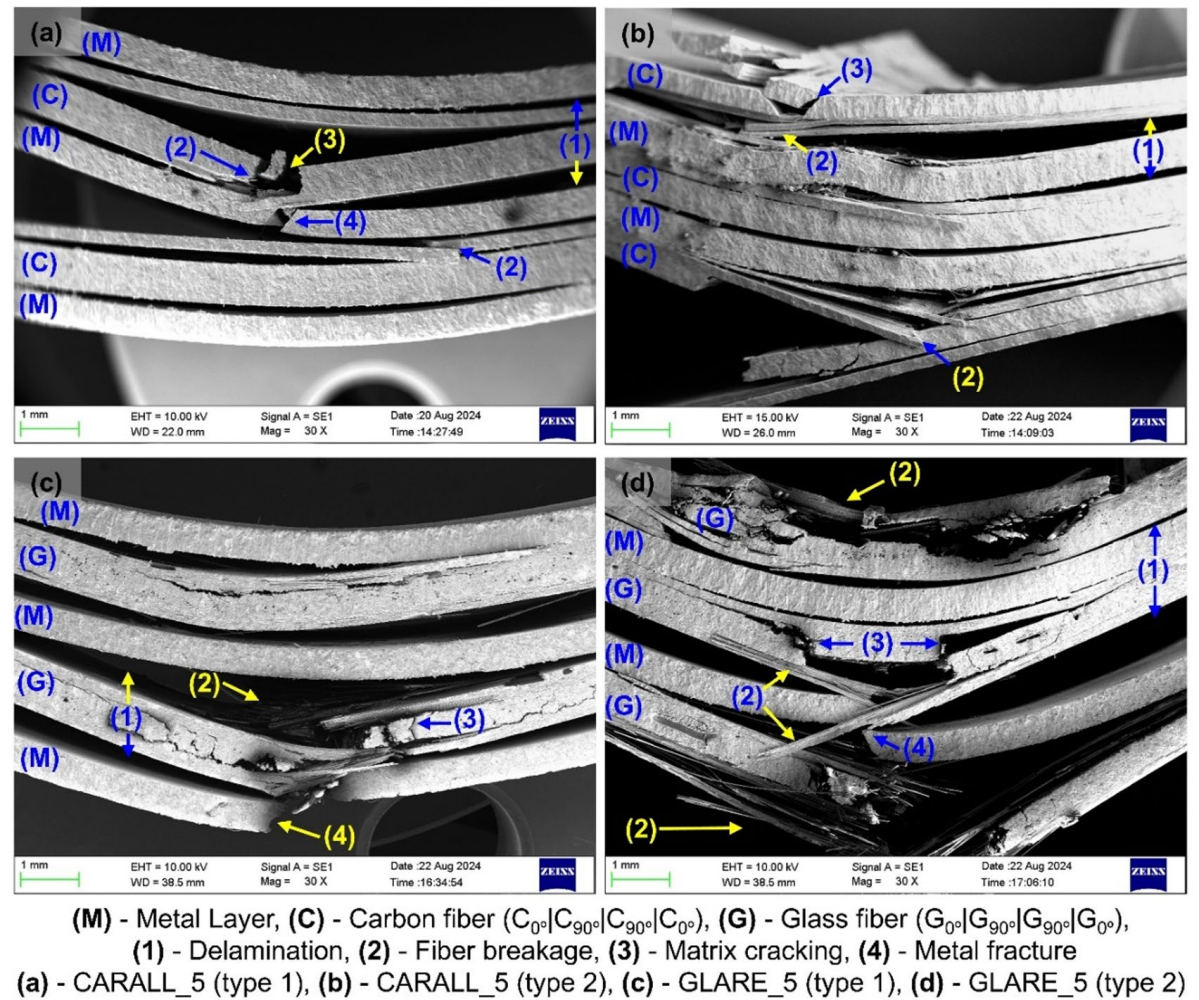
SEM images of failed FMLs under flexural loading.

Figure 11a presents a visual depiction of the failed type-1 and type-2 CARALL FMLs, while Fig. 12a, b displays microscale images from the SEM analysis. The substantial load-sharing ability of carbon fibers significantly alleviates the stress on the metal layers. In type-1 CARALL FML, the upper layer of aluminum on the compressive side experiences compressive yielding and buckling, as illustrated in Fig. 12a. In contrast, in type-2 CARALL FML, where the carbon fiber layer is positioned on the top side, compressive yielding can initiate interfacial delamination, potentially leading to the fracture of the top aluminum layer, as depicted in Fig. 12b. In both type-1 and type-2 CARALL FMLs, the aluminum layers located on the lower side undergo substantial tensile yielding, which leads to a permanent fracture in the case of type-2 CARALL FML.

Additionally, top carbon fiber layers are subjected to compressive loading during flexural tests. As a result, the fiber composite layer experiences matrix crushing as seen in Fig. 12a, b. As illustrated in Fig. 12b, the lower fiber layers of type-2 CARALL, when subjected to tensile stress, exhibit matrix cracking. Moreover, both CARALL_5 FMLs exhibited fiber breakage and delamination in their fiber layers, as clearly illustrated in Fig. 12a, b.

The type-1 and type-2 GLARE_5 FMLs exhibited damage akin to that of CARALL-5 FMLs, but the damage was significantly more extensive, as illustrated in Fig. 11b. GLARE_5 is composed of glass fiber/epoxy layers, which have a lower elastic modulus and compressive strength compared to the carbon fiber/epoxy layers found in CARALL_5. In GLARE_5, the reduced stiffness of glass fibers results in increased elastic and plastic deformation, leading to matrix cracking and significant interfacial stresses at the aluminum–composite junctions. This encourages fiber fracture and delamination (see Fig. 12c, d which hastens damage to progress under flexural loads. Moreover, aluminum layers at the top side showed signs of yielding and buckling in the case of type-1 GLARE, while permanent fracture in the case of type-2 GLARE FML. Whereas the bottom side aluminum layers in the case of type-1 and type-2 GLARE showed substantial tensile yielding with permanent fracture, as seen in Fig. 12c, b.

**Fig. 13:**
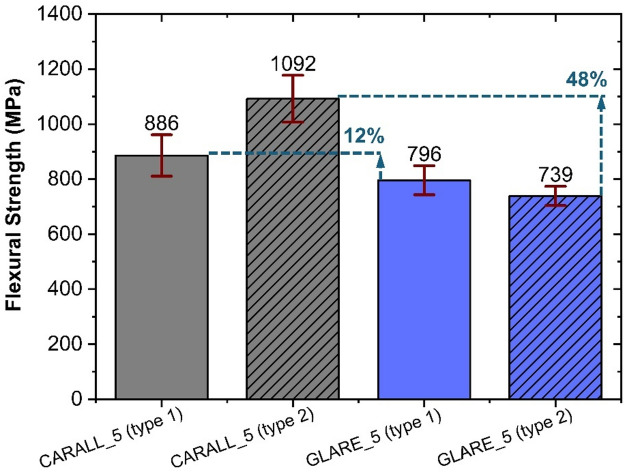
Flexural strength of the developed FMLs.

To gain a deeper insight into the flexural behavior of the developed FMLs, the flexural strength of type-1 and type-2 CARALL_5 and GLARE_5 FMLs was measured and quantified, as depicted in Fig. 13. Both type-1 and type-2 CARALL_5 FMLs demonstrated higher flexural strength than the GLARE_5 FMLs. The average flexural strength of type-1 CARALL_5 FML was 886 ± 75 MPa, which is 12% greater than that of the type-1 GLARE_5 FML (796 ± 52 MPa). Similarly, type-2 CARALL_5 FML achieved a flexural strength of 1092 ± 85 MPa, surpassing its GLARE counterpart by 48%. Moreover, the highest flexural strength, 1092 MPa, was achieved by type-2 CARALL_5 FML, which is 23% higher than that of type-1 CARALL_5 FML (739 ± 34 MPa). The enhanced flexural strength of CARALL_5 compared to GLARE_5 is due to the superior load-sharing and bonding capabilities of carbon fibers over glass fibers.

**Fig. 14:**
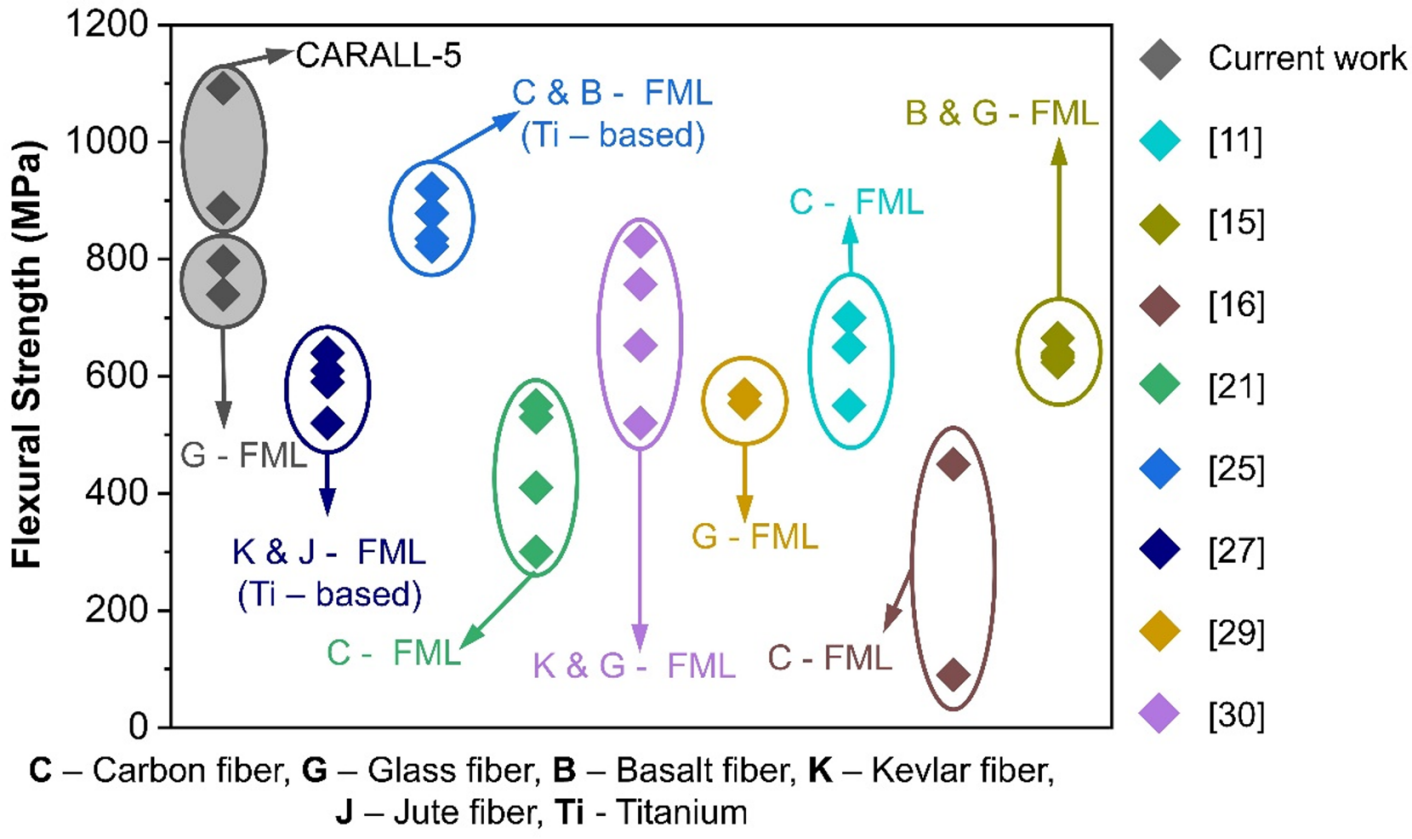
Comparative analysis for flexural strength of CARALL_5 with previous studies.

Additionally, Fig. 14 illustrates a comparison of the flexural strength values of the developed FMLs with those reported in existing studies. The comparison included all aluminum-based FMLs reported in the literature, as well as recent findings on titanium-based FMLs. Furthermore, the detailed summary of the literature on flexural analysis is provided in Table S2 of the supplementary information. The flexural strength of the developed CARALL_5 FMLs surpasses the strength values reported in previously published literature^11,15,16,21,25,27,29,30^. Therefore, the developed type-2 CARALL_5 FML is suitable for applications where resistance towards bi-axial loading is necessary.

### Impact energy analysis

**Fig. 15:**
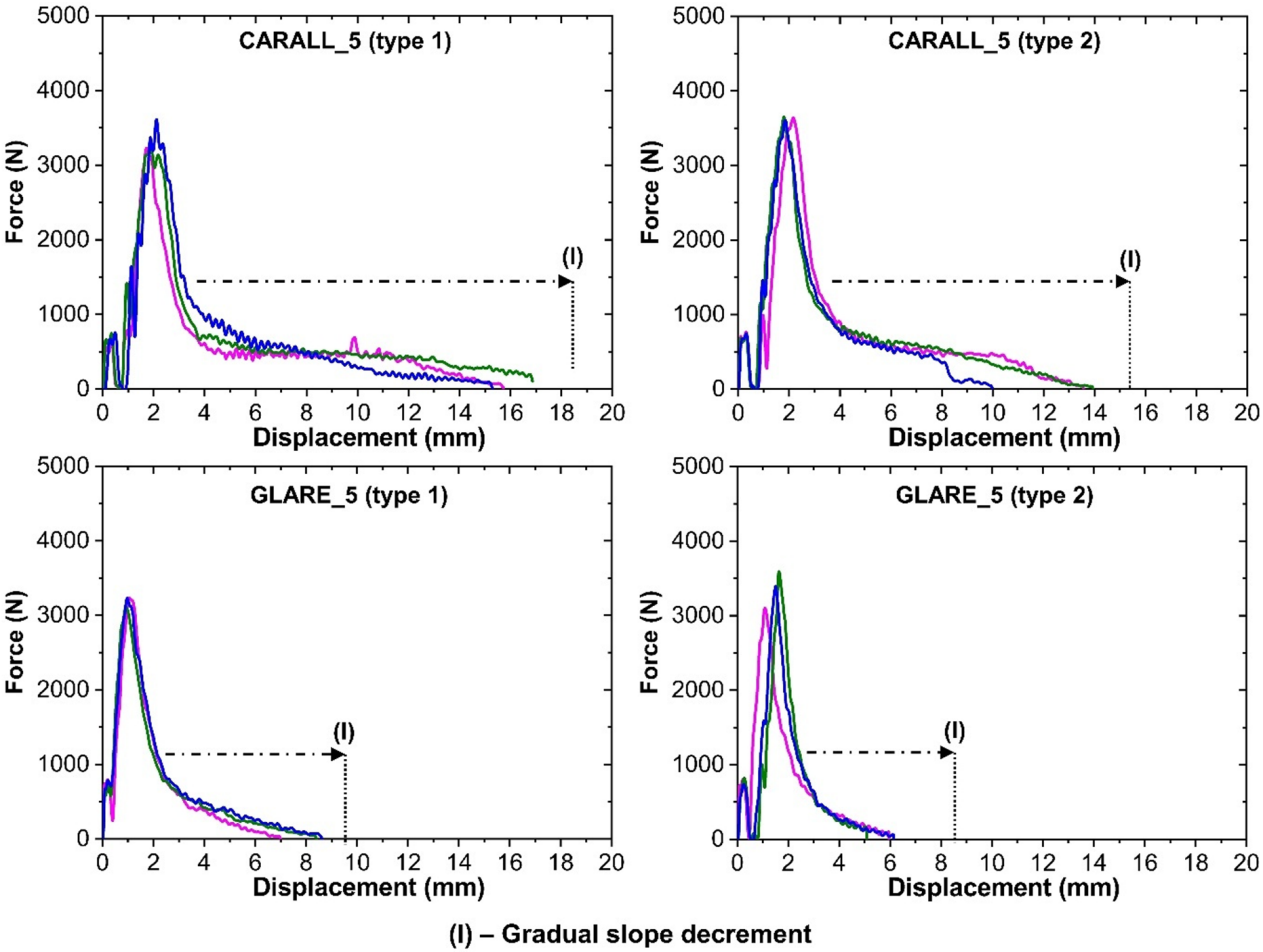
Force versus displacement curves of CARALL_5 and GLARE_5 from the Charpy impact test.

Figure 15 shows the impact force versus displacement graphs for the type-1 and type-2 CARALL and GLARE FMLs, and the supporting force versus time curves are provided in Fig. S1 of supplementary information. The impact energy curve, represented through force–displacement, provides insight into the impact response of FMLs, as it illustrates how energy is dissipated throughout the impact event. The impact energy curve typically exhibits a distinct two-stage behavior, reflecting the hybrid response of metal and composite layers. In the initial stage, the curve shows a steep and nearly linear rise in force, corresponding to elastic deformation of the laminate as it begins to absorb impact energy. This is followed by the attainment of a peak force. During this phase, the metal layers undergo plastic deformation, contributing significantly to energy absorption. Subsequently, the curve transitions into a gradually decreasing slope, where continued plastic deformation of the metal layers and progressive failure of the fiber composite due to fiber–matrix debonding, matrix cracking, and delamination at the fiber–metal interfaces dominate the response, allowing the laminate to absorb additional energy prior to complete failure. Overall, the area enclosed under the curve represents the total energy absorbed by the CARALL and GLARE FMLs during the impact event. Figure 15 also shows the influence of the fiber type and stacking sequence on the impact energy. The Charpy impact test conducted on CARALL FMLs demonstrates that they absorb a considerable amount of impact energy, as evidenced by the energy curve, which shows a larger, more gradually decreasing slope compared to that of GLARE FMLs. The behaviour is attributed to the increased volume of fiber matrix debonding. The fibers debonding from the matrix provides further scope to dissipate energy, thereby resulting in higher impact strength of the laminate. Debonding extends the scope of energy absorption, increasing the area under the force–displacement curve due to retarded progression towards complete failure^20,31^. Fiber matrix debonding is induced because of the difference in strength values between fibers and the polymer matrix. The difference in the fiber and matrix strength is higher in the case of CARALL_5 in comparison with GLARE_5. Therefore, CARALL_5 FMLs showcased a more gradual decrement in the force curve, subsequently resulting in increased energy absorption.

Furthermore, as illustrated in Fig. 15, type-1 CARALL and GLARE FMLs exhibit a prolonged force–displacement response compared to type-2 FMLs, suggesting a slower and more gradual failure process. This extended duration for the type-1 FMLs is attributed to the higher metal volume fractions in this configuration. The metallic component experiences considerable plastic deformation before fracture, extending the duration of contact. Additionally, this is indicative of more efficient fiber–matrix debonding, matrix cracking, and delamination, resulting in improved impact resistance. Figure 15 demonstrates that the peak impact forces are greater for CARALL FMLs compared to GLARE FMLs. CARALL FML is composed of carbon fiber–reinforced polymer layers, which have a much higher tensile strength and elastic modulus than the glass fiber reinforcements used in GLARE FML. This increased stiffness results in CARALL FML experiencing less local deformation when subjected to impact, causing the force applied to increase more quickly and reach a higher value. Conversely, the lower stiffness of glass fibers allows for more deformation, resulting in lower peak forces. Additionally, the strong load-carrying capability of carbon fibers delays the onset of major damage mechanisms like fiber fracture and extensive delamination. This allows CARALL FML to endure higher loads before significant stiffness degradation occurs, which is reflected in higher peaks on the impact force–displacement curves. Although CARALL FML may experience significant fiber–matrix debonding due to the greater property mismatch at the interface, this damage occurs after the peak force is reached and mainly contributes to energy absorption rather than reducing the peak load.

**Fig. 16:**
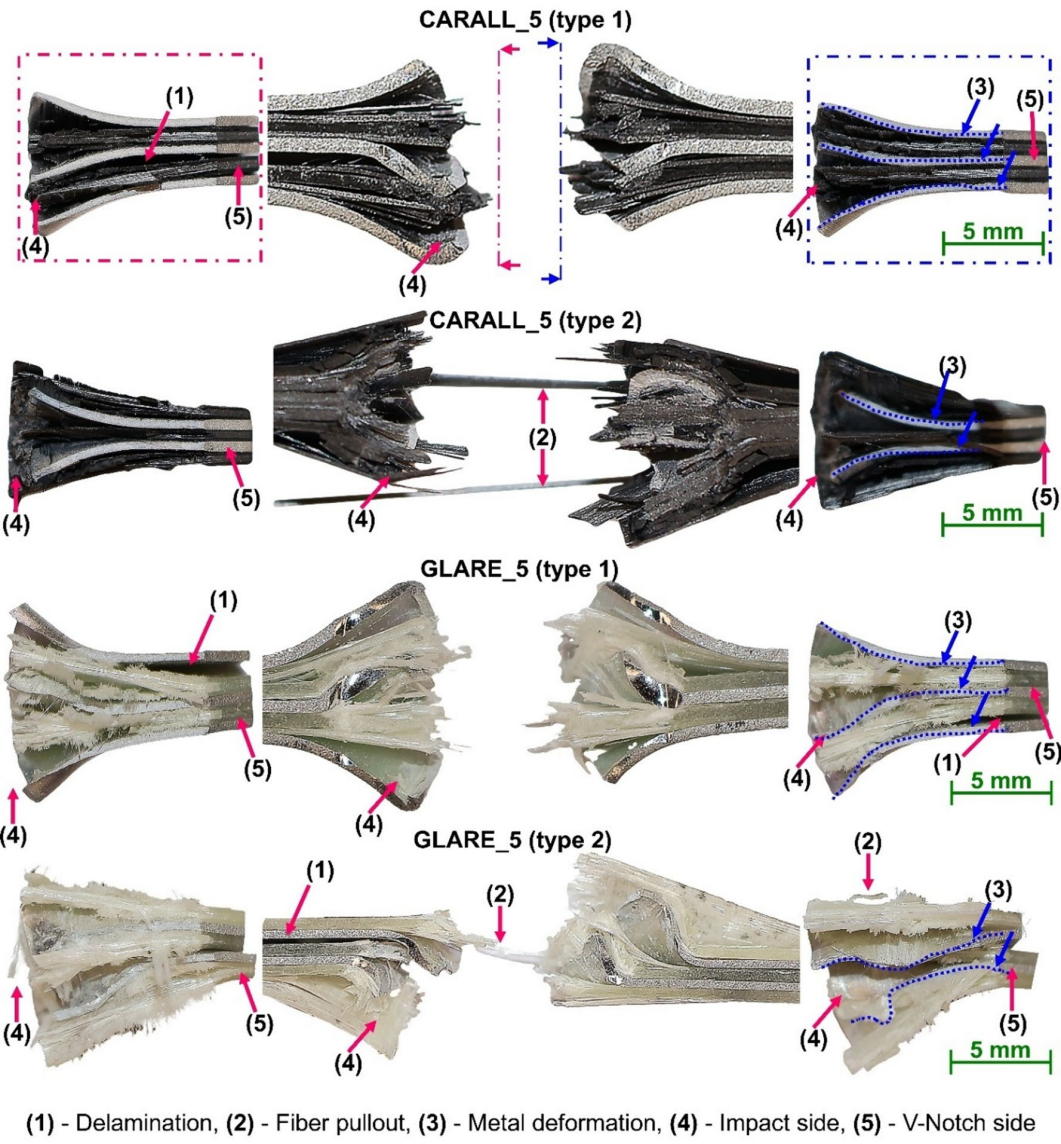
Pictorial views of failed FMLs under impact loading.

**Fig. 17:**
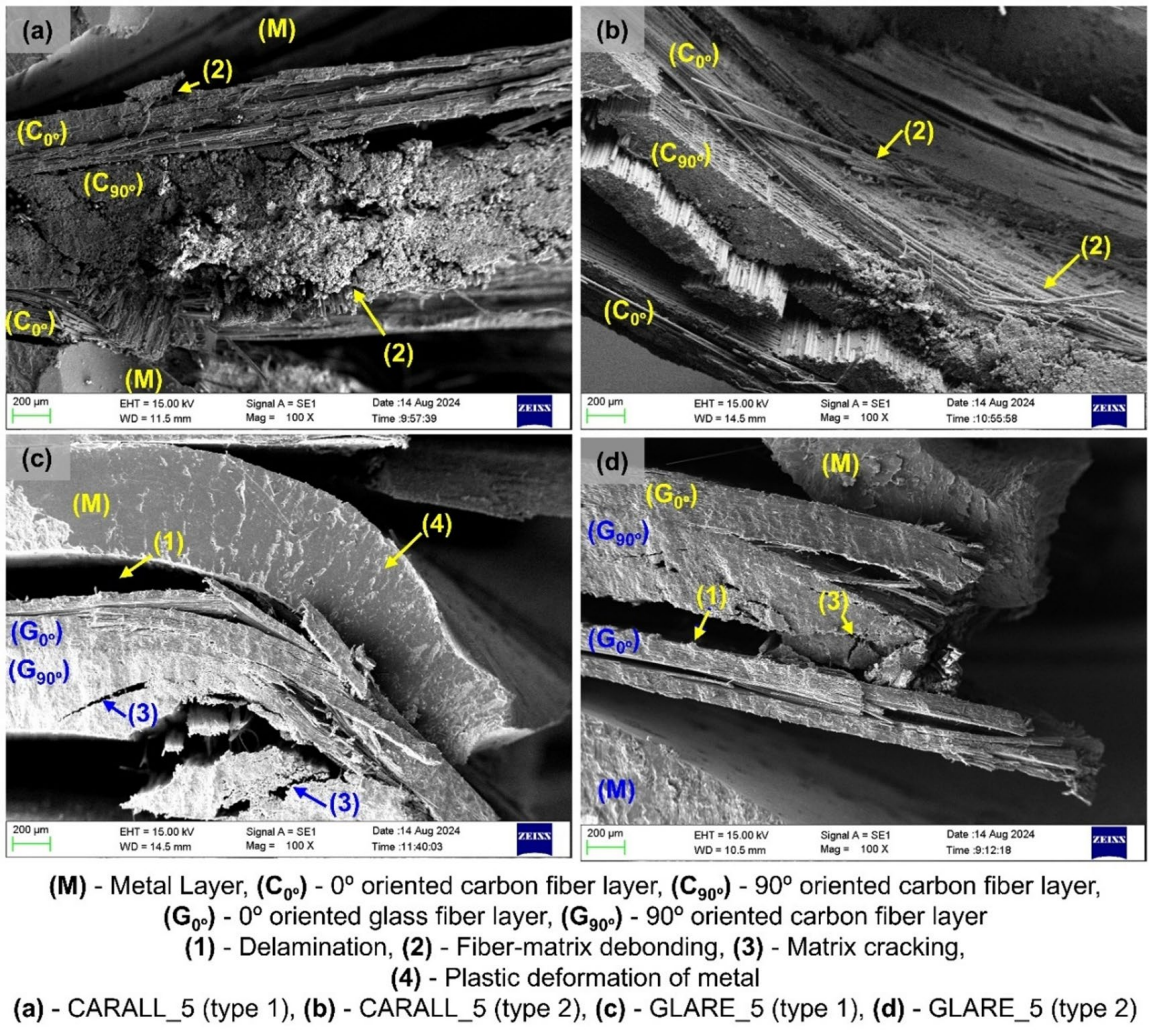
SEM images of failed laminates under impact loading.

To explore the impact behavior of the developed FMLs, an examination of the failed specimens was performed. Figure 16 illustrates the various mechanisms through which FMLs experienced failure when subjected to impact loading, and Fig. 17 offers microscale images of these failed samples captured via scanning electron microscopy. During charpy impact tests, FML specimens experienced a complete transverse fracture at the notch, which suggests plastic deformation, fiber–matrix debonding, matrix cracking, and delamination, typically indicating greater energy absorption. During impact loading, the presence of a notch creates a strong stress concentration. The area adjacent to the notch endures the highest tensile stress, which facilitates the initiation of cracks. Once a crack begins, it progresses through the specimen towards the neutral axis, where tensile and compressive stresses are in equilibrium. As the crack continues to extend, it encounters a zone dominated by compressive stress, which decelerates and eventually halts its growth. This stress gradient governs the crack path and final fracture behavior during impact loading^23^. At the moment of impact, the composite layer where the fibers are oriented at 0° near the notch are subjected to tensile load leading to fiber–matrix debonding, while the composite layer where the fibers are oriented at 90° show matrix cracking, initiating the damage in the fiber-reinforced composite layers. As the load increases, the impact energy is mainly absorbed through composite layer which undergoes fiber–matrix debonding. From Figs. 16 and 17, it can be seen that the metal layer in FMLs were subjected to severe plastic deformation before shearing. Additionally, the failed FMLs showed signs of interfacial delamination, which further helped in delaying the failure of the FMLs.

**Fig. 18:**
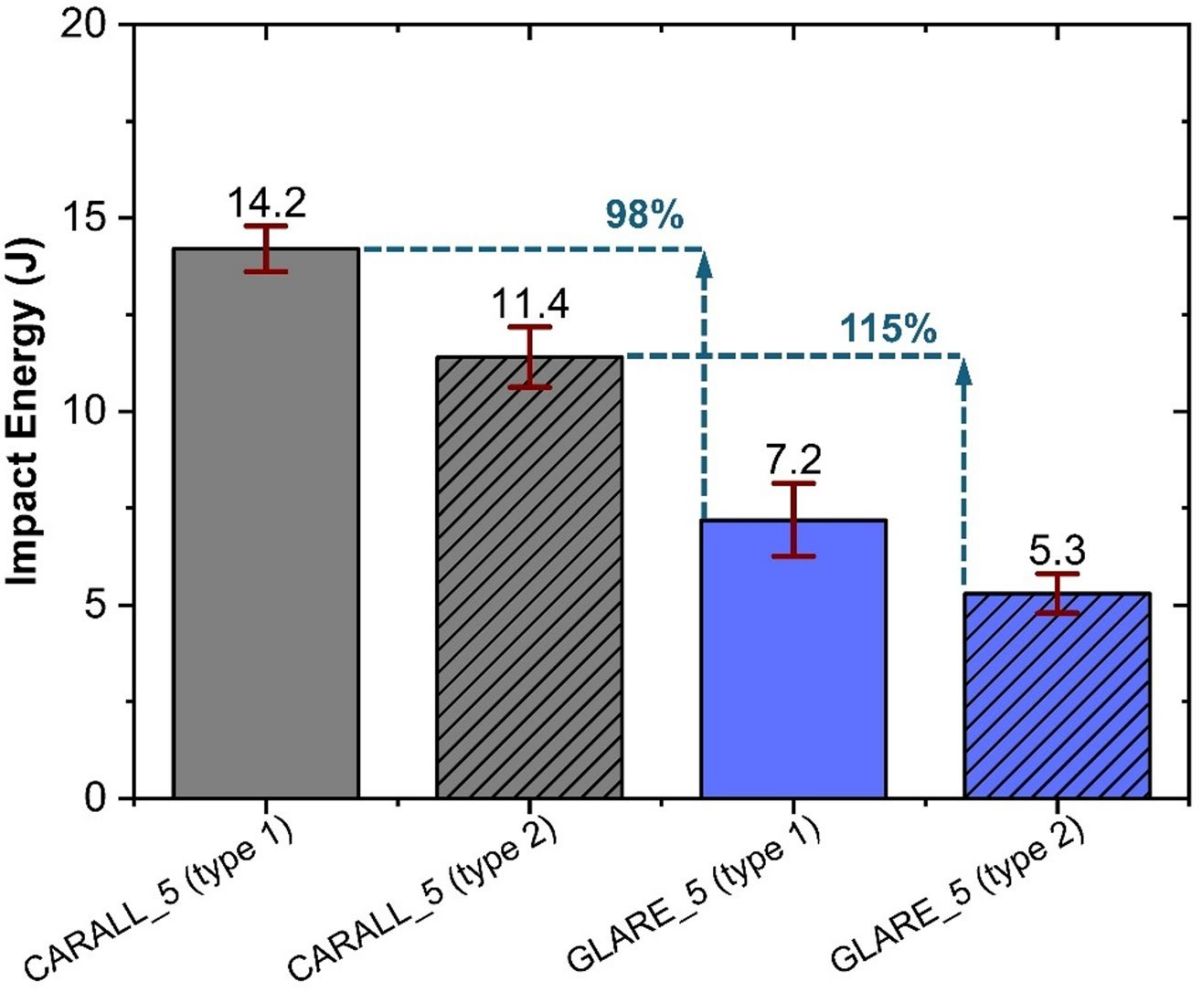
Impact energies exhibited by the developed FMLs.

Furthermore, the impact energy of all the developed FMLs is presented in Fig. 18. In general, CARALL_5 FMLs displayed the capacity to withstand significantly higher impact energy than GLARE_5 FMLs. The type-1 CARALL_5 FML displayed an average impact energy of 14.2 ± 0.59 J, which is 98% higher than that of type-1 GLARE_5 FML. Whereas type-2 CARALL_5 FML resulted in an average impact energy of 11.4 ± 0.78 J, which is 115% higher in comparison with the type-2 GLARE_5 FML (see Fig. 18). As discussed, carbon fibers possess significantly higher tensile strength and stiffness compared to glass fibers, while the polymer matrix remains the same. This leads to higher interfacial stress concentration and increased tendency for fiber–matrix debonding in CARALL relative to GLARE, thus enhancing energy dissipation. Additionally, the strong load-carrying capability of carbon fibers delays the onset of major damage mechanisms such as fiber fracture and extensive delamination. This enables CARALL5 to sustain higher energies before significant stiffness degradation occurs. Moreover, the impact energies for type-2 CARALL and GLARE FMLs were measured at 11.4 ± 0.78 J for CARALL_5 and 5.3 ± 0.51 J for GLARE_5, which are notably lower than those of type-1 CARALL_5 and GLARE_5 FMLs, recorded at 14.2 ± 0.59 J and 7.2 ± 0.94 J, respectively. This increase in energy absorption in type-1 FMLs is attributed to a higher metal volume fraction. The failure analysis indicated that the aluminum layers played a significant role in absorbing impact energy. However, the higher fiber volume fractions and the brittle nature of type-2 FMLs restricted the aluminum’s contribution to energy absorption, resulting in a 24% and 35% decrease in absorbed impact energy for type-2 CARALL_5 and GLARE_5, respectively. This implies that positioning an aluminum layer on the exterior in the stacking sequence is beneficial under impact loading, enhancing the impact strength of both FMLs.

**Fig. 19:**
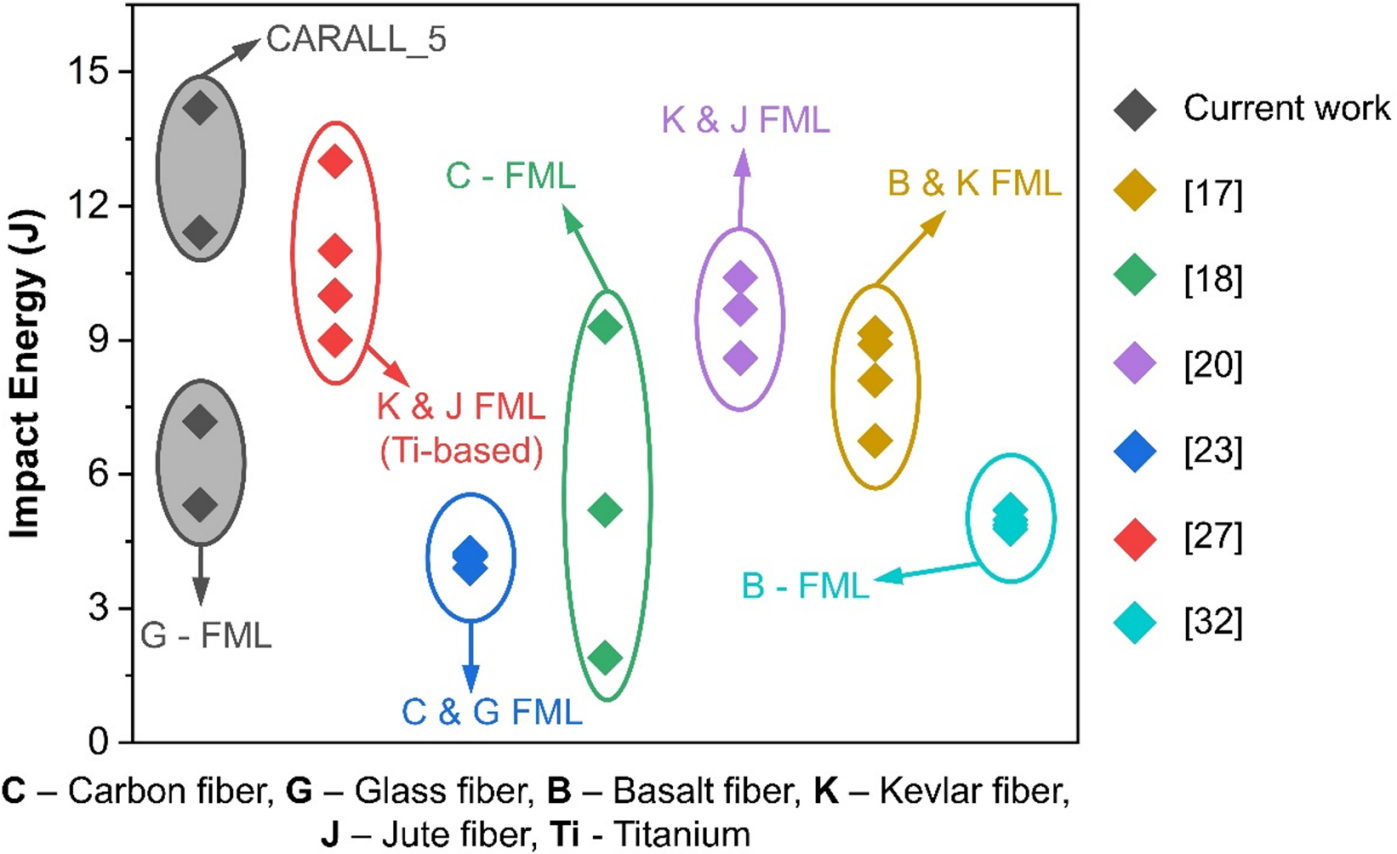
Comparative analysis for flexural strength of CARALL_5 with previous studies.

The impact energy of the developed FMLs were compared with the impact energies of aluminum-based FMLs available in the literature^17,18,20,23,32^, and the analysis is presented as Fig. 19. Additionally, recent research on titanium-based FMLs was included for comparison purposes. Additionally, the detailed summary of the literature pertaining to impact studies on FMLs is provided in Table S3 of the supplementary information. As shown in Fig. 19, the comparative analysis confirms that the newly developed CARALL_5 FMLs demonstrated superior impact strength compared to other FMLs found in the literature. Therefore, the developed CARALL_5 is a viable option for impact applications, where materials with higher impact resistance are required.

## Conclusions

The study presented the experimental evaluation of developed CARALL_5 and GLARE_5 FMLs with two distinct stacking sequences under tensile, flexural, and impact loading conditions. The aluminium layers constituted as the outer plies in type-1 (A|C|A|C|A) stacking sequence, and in type-2 (C|A|C|A|C) sequence, fiber layers formed the outer surfaces. The following conclusions can be drawn:

- Under tensile loading conditions, type 2 CARALL_5 FMLs exhibited the highest tensile strength of 776 ± 73 MPa, which is 79.7% higher than GLARE_5 FMLs (466 ± 39 MPa). The higher tensile strength of CARALL_5 FMLs is attributed to the high strength of carbon fibers. Type-1 CARALL_5 FMLs displayed comparatively lower tensile strength of 610 ± 53 MPa. However, type-1 laminates showcased a stepped failure pattern during tensile loading indicated a gradual progression of damage, enhancing the endurance of the laminate.
- When subjected to tensile stress, type-1 CARALL_5 and GLARE_5 FMLs exhibited failure modes such fiber breakage, matrix cracking, and delamination. In contrast, type-2 CARALL_5 FML experienced concurrent failure of both the metal and fiber-reinforced composite layers, characterized by fiber pull-out, matrix cracking, and significant delamination. The aluminum layers in type-1 FMLs underwent significant elongation before fracture, while type-2 FMLs displayed limited the plastic deformation before fracture.
- The maximum flexural strength of 1092 ± 85 MPa was observed in type-2 CARALL_5 FMLs, which is 48% higher than type-2 GLARE_5 FMLs. The improved flexural strength is due to the higher stiffness and elastic modulus of carbon fibers. The flexural stress–strain curve for the type-1 FMLs exhibit slope with pronounced strain, indicating yielding of the metal layers. Whereas in type-2 FMLs, a minor change in the trajectory and slope of the curves was observed, which is attributed to the higher fiber volume fraction.
- Under flexural loading condition, in type-1 FMLs, the upper layer of aluminum on the compressive side underwent compressive yielding and buckling, whereas, in type-2 CARALL FML, where the carbon fiber layer is positioned on the top side, compressive yielding triggered interfacial delamination, resulting in the fracture of the top aluminum layer. In both type-1 and type-2 FMLs, the aluminum layers on the tensile side underwent substantial tensile yielding, resulting in a permanent fracture in type-2 CARALL FML.
- CARALL_5 type-1 FMLs exhibited a superior impact energy absorption of 14.2 ± 0.59 J, which is 98% higher than GLARE_5 FMLs (7.2 ± 0.94 J). Prominent fiber-matrix debonding and tearing of aluminium sheets due to superior interfacial bonding were the key failure mechanisms that contributed to the enhanced impact strength of CARALL_5. Also, the type-1 FMLs absorbed higher impact energy than the type-2 FMLs because of increased metal volume fraction.
- Under impact loading condition, both CARALL and GLARE FMLs displayed signs of plastic deformation, fiber–matrix debonding, matrix cracking, and delamination. However, CARALL FML demonstrated a higher capacity for energy absorption.

According to the results of the current study, type-1 CARALL_5 FMLs are best suited for applications involving high tensile and impact loads, where residual load bearing even after damage is critical. Conversely, type-2 CARALL_5 FMLs can be considered where endurance against bi-axial loading is necessary.

## Supplementary Information

Below is the link to the electronic supplementary material.

Supplementary Material 1

## Data Availability

The datasets generated during and/or analyzed during the current study are available from the corresponding author upon reasonable request.
