# Supplementary material for: Effect of stacking sequence on the mechanical behaviour of CARALL and GLARE fiber metal laminates
Source: Sci Rep. 2026 Apr 24;16:18844. doi: 10.1038/s41598-026-50170-5 (PMC13273083; doi:10.1038/s41598-026-50170-5)
Supplement: Supplementary file 1 — Supplementary Material 1 [file 41598_2026_50170_MOESM1_ESM.docx]

**Supplementary Information**

**Effect of Stacking Sequence on the Mechanical Behaviour of CARALL and GLARE Fiber Metal Laminates**

Madhusudhan Balkundhi, Satish Shenoy Baloor, and Gururaj Bolar*

Manipal Institute of Technology, Manipal Academy of Higher Education, Manipal, India

***** Correspondence: [gururaj.bolar@manipal.edu](mailto:gururaj.bolar@manipal.edu)

**Manuscript ID:** b91a9b46-1b4e-4dc4-8c9d-ed60a8f8ec3e

Table S.1. Summary of literature comparison for tensile strength.

| Reference | Material | ASTM | Fiber Volume Fraction (%) | Thickness (mm) | Stacking sequence studies | Tensile strength (MPa) |
| --- | --- | --- | --- | --- | --- | --- |
| Present work | Aluminum with carbon and glass fiber | D 638 | 54 | 3.21 | A\|C\|A\|C\|A,  C = C0°\|C90°\|C90°\|C0° | 887.45 |
|  |  |  | 72 | 3.31 | C\|A\|C\|A\|C,  C = C0°\|C90°\|C90°\|C0° | 1091.72 |
|  |  |  | 54 | 3.2 | A\|G\|A\|G\|A,  G = G0°\|G90°\|G90°\|G0° | 796.18 |
|  |  |  | 73 | 3.32 | G\|A\|G\|A\|G,  G = G0°\|G90°\|G90°\|G0° | 739.41 |
| [1] | Titanium with Kevlar and jute fibers | D 3039 | - | ~ 2.6 | 2/1 FML | ~ 450 |
|  |  |  |  | ~ 4.3 | 3/2 FML | ~ 600 |
|  |  |  |  | ~ 5.9 | 4/3 FML | ~ 750 |
|  |  |  |  | ~ 7.5 | 5/4 FML | ~ 780 |
| [2] | Titanium with carbon and basalt fibers | D 3039 | 50.76 | 2.6 | T\|B\|B\|C\|C\|C\|C\|B\|B\|T | 474.32 |
|  |  |  |  |  | T\|C\|C\|B\|B\|B\|BC\|C\|T | 456.68 |
|  |  |  |  |  | T\|C\|C\|B\|B\|C\|C\|B\|BT | 553.13 |
|  |  |  |  |  | T\|C\|B\|C\|B\|C\|B\|C\|B\|C\|B\|T | 545.72 |
| [3] | Aluminum with carbon and glass fibers | D 3039 | 100 | - | H-FRP G\|C\|G\|G\|C\|G | 810 |
|  |  |  | 81 |  | H-FML G\|C\|G\|A\|G\|C\|G | 765 |
|  |  |  | 69 |  | H-FML A\|G\|C\|G\|G\|C\|G\|A | 747.8 |
|  |  |  | 60 |  | H-FML A\|G\|C\|G\|A\|G\|C\|G\|A | 734 |
| [4] | Aluminum with carbon fibers | D 638 | 49.65 | 4 | Type 1- A\|C\|A\|C\|A (twill weave) | 192.92 |
|  |  |  |  |  | Type 2- C\|A\|C\|A (twill weave) | 155.83 |
|  |  |  |  |  | Type 3- C\|C\|C\|C\|C\|C\|C (twill weave) | 145.83 |
| [5] | Aluminum with Kevlar and jute fibers | D 638 | 27.3 | 2.75 | A\|K\|A\|K\|A\|K\|A | 320.5 |
|  |  |  | 46.7 | 3.75 | A\|J\|A\|K\|A\|J\|A | 315.4 |
|  |  |  | 42.9 | 3.5 | A\|K\|J\|A\|J\|K\|A | 325.6 |
| [6] | Aluminum with glass fibers | D 3039 | - | 3.2 | GLARE - cross ply 0\|90 | 295 |
|  |  |  |  |  | GLARE - Woven fiber glass | 319 |
| [7] | Aluminum with carbon fibers | D 3039 | - | 3.5 | SS1 - A\|C_11_\|A | 500 |
|  |  |  |  |  | SS2 - A\|C_2_\|A\|C\|A\|C\|A\|C_2_\|A | 420 |

Table S.2. Summary of literature comparison for flexural strength.

| Reference | Material | ASTM | Fiber Volume Fraction (%) | Thickness (mm) | Material and stacking sequence | Flexural Strength (MPa) |
| --- | --- | --- | --- | --- | --- | --- |
| Present work | Aluminum with carbon and glass fiber | D 790 | 54 | 3.21 | A\|C\|A\|C\|A,  C = C0°\|C90°\|C90°\|C0° | 887.45 |
|  |  |  | 72 | 3.31 | C\|A\|C\|A\|C,  C = C0°\|C90°\|C90°\|C0° | 1091.72 |
|  |  |  | 54 | 3.2 | A\|G\|A\|G\|A,  G = G0°\|G90°\|G90°\|G0° | 796.18 |
|  |  |  | 73 | 3.32 | G\|A\|G\|A\|G,  G = G0°\|G90°\|G90°\|G0° | 739.41 |
| [1] | Titanium with Kevlar and jute fibers | D 790 | - | ~ 2.6 | 2/1 FML | ~ 640 |
|  |  |  |  | ~ 4.3 | 3/2 FML | ~ 610 |
|  |  |  |  | ~ 5.9 | 4/3 FML | ~ 590 |
|  |  |  |  | ~ 7.5 | 5/4 FML | ~ 520 |
| [2] | Titanium with carbon and basalt fibers | D 7264 | 50.76 | 2.6 | T\|B\|B\|C\|C\|C\|C\|B\|B\|T | 834.66 |
|  |  |  |  |  | T\|C\|C\|B\|B\|B\|BC\|C\|T | 821.95 |
|  |  |  |  |  | T\|C\|C\|B\|B\|C\|C\|B\|BT | 878.27 |
|  |  |  |  |  | T\|C\|B\|C\|B\|C\|B\|C\|B\|C\|B\|T | 920.48 |
| [8] | Aluminum with carbon fiber | D 7264 | - | - | A\|C\|A | 300 |
|  |  |  |  |  | A\|C\|A\|C\|A | 550 |
|  |  |  |  |  | A\|C\|A - with adhesive | 530 |
|  |  |  |  |  | A\|C\|A\|C\|A with adhesive | 410 |
| [9] | aluminum with Kevlar and glass fibers | D 790 | 25 | 1.85 | 0 Wt % Graphene nanoparticles | 757 |
|  |  |  | 25 | 1.83 | 0.1 Wt % Graphene nanoparticles | 830 |
|  |  |  | 24 | 1.87 | 0.25 Wt % Graphene nanoparticles | 653 |
|  |  |  | 24 | 1.79 | 0.5 Wt % Graphene nanoparticles | 520 |
| [6] | Aluminum with glass fiber | D 790 | - | 3.2 | GLARE - cross ply 0\|90 | 1125 |
|  |  |  |  |  | GLARE - Woven fiber glass | 569 |
| [10] | Aluminum with carbon fiber | D 790 | - | - | Only CAA Al\|0\|Al\|0\|Al | 900 |
|  |  |  |  |  | Only CAA Al\|90\|Al\|0\|Al | 850 |
|  |  |  |  |  | Only CAA Al\|90\|Al\|90\|Al | 500 |
|  |  |  |  |  | Only MA(220) Al\|0\|Al\|0\|Al | 1280 |
|  |  |  |  |  | Only MA(220) Al\|90\|Al\|0\|Al | 1200 |
|  |  |  |  |  | Only MA(220) Al\|90\|Al\|90\|Al | 800 |
|  |  |  |  |  | 220+CAA Al\|0\|Al\|0\|Al | 1150 |
|  |  |  |  |  | 220+CAA Al\|90\|Al\|0\|Al | 1000 |
|  |  |  |  |  | 220+CAA Al\|90\|Al\|90\|Al | 800 |
| [11] | Aluminum with carbon fiber | D 790 | - | 5 | SS1- C\|A\|C (with adhesive) | 650 |
|  |  |  |  |  | SS2- C\|A\|C (without adhesive) | 700 |
|  |  |  |  |  | SS3- C\|A\|C\|A\|C (with adhesive) | 550 |
|  |  |  |  |  | SS4- C\|A\|C\|A\|C (without adhesive) | 650 |
| [7] | Aluminum with carbon fiber | D 3410 M | - | 3.5 | SS1 - Al\|C_11_\|Al | 450 |
|  |  |  |  |  | SS2 - Al\|C_2_\|Al\|C\|Al\|C\|Al\|C_2_\|Al | 90 |
| [12] | Aluminum with glass fiber | - | - | 1.54 | 3/2 - 0\|0 | 1189.51 |
|  |  |  |  | 1.54 | 3/2 - 0\|90 | 1198.32 |
|  |  |  |  | 2.18 | 4/3 - 0\|0 | 1202.64 |
|  |  |  |  | 2.18 | 4/3 - 0\|90 | 1203.29 |
|  |  |  |  | 2.85 | 5/4 - 0\|0 | 868.53 |
|  |  |  |  | 2.85 | 5/4 - 0\|90 | 838.44 |
|  |  |  |  | 3.36 | 6/5 - 0\|0 | 779.46 |
|  |  |  |  | 3.36 | 6/5 - 0\|90 | 757.75 |
| [13] | Aluminum with basalt and glass fiber | D 790 | 25 | 1.85 | A\|B\|B\|B\|B\|A | 665 |
|  |  |  | 26 | 1.8 | A\|B\|G\|G\|B\|A | 640 |
|  |  |  | 26 | 1.82 | A\|B\|G\|B\|G\|A | 631.5 |
|  |  |  | 26 | 1.8 | A\|G\|B\|B\|G\|A | 624 |
|  |  |  | 23 | 1.78 | A\|G\|G\|G\|G\|A | 635 |


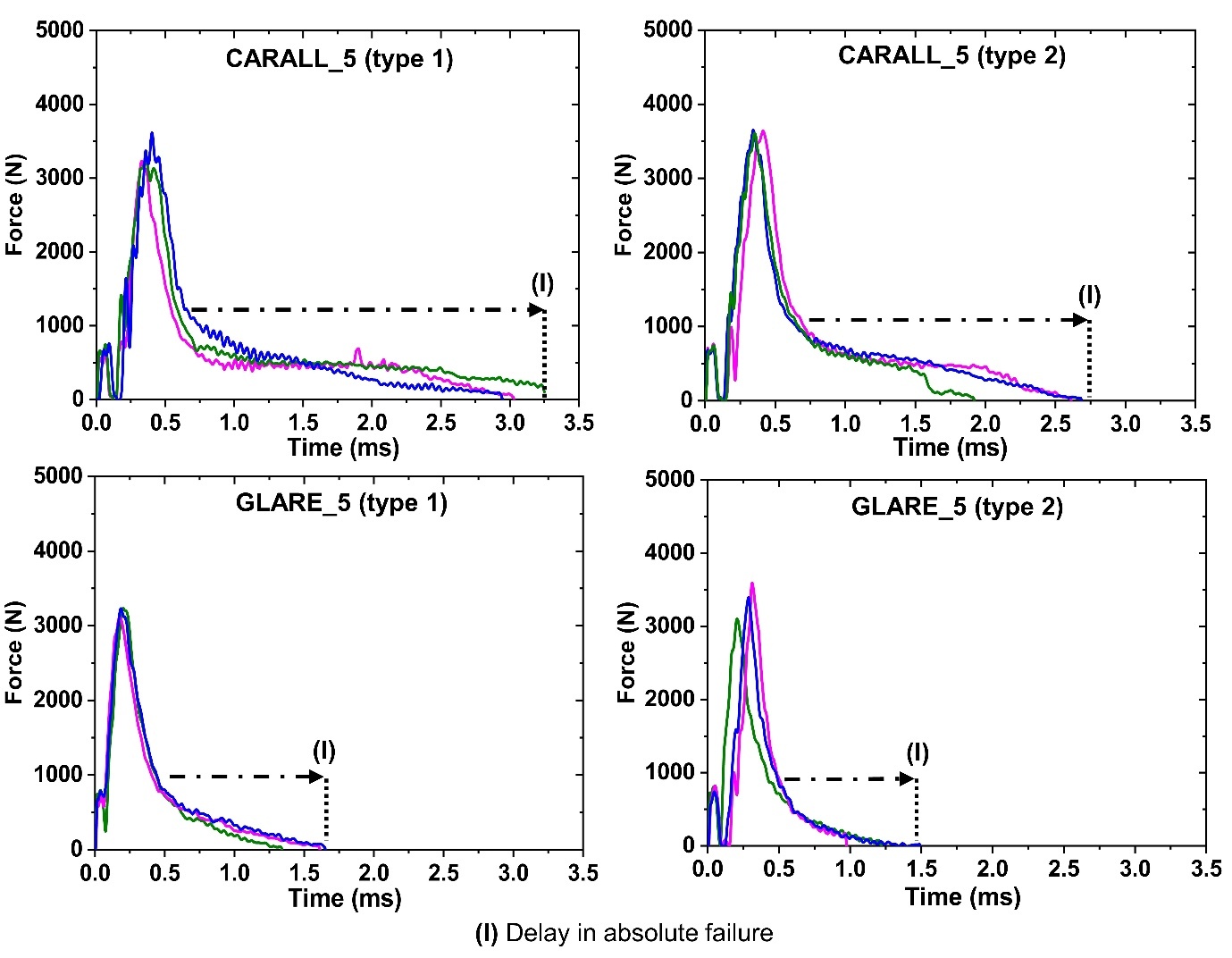


Figure S.1. Force vs. time curves of CARALL_5 and GLARE_5 from the Charpy impact test.

Table S.3. Summary of literature comparison for impact energy.

| Reference | Material | ASTM | Fiber Volume Fraction (%) | Thickness (mm) | Material and stacking sequence | Impact energy (J) |
| --- | --- | --- | --- | --- | --- | --- |
| Present work | Aluminum with carbon and glass fiber | D 256 | 54 | 3.21 | A\|C\|A\|C\|A,  C = C0°\|C90°\|C90°\|C0° | 14.2 |
|  |  |  | 72 | 3.31 | C\|A\|C\|A\|C,  C = C0°\|C90°\|C90°\|C0° | 11.41 |
|  |  |  | 54 | 3.2 | A\|G\|A\|G\|A,  G = G0°\|G90°\|G90°\|G0° | 7.18 |
|  |  |  | 73 | 3.32 | G\|A\|G\|A\|G,  G = G0°\|G90°\|G90°\|G0° | 5.32 |
| [1] | Titanium with Kevlar and jute fibers | A 370 | - | ~ 2.6 | 2/1 FML | ~ 9 |
|  |  |  |  | ~ 4.3 | 3/2 FML | ~ 10 |
|  |  |  |  | ~ 5.9 | 4/3 FML | ~ 11 |
|  |  |  |  | ~ 7.5 | 5/4 FML | ~ 13 |
| [14] | Aluminum with carbon and glass fibers | E 23 | - | - | SS1 - A\|G\|G\|G\|A | 3.9 |
|  |  |  |  |  | SS2 - A\|G\|G\|G\|G\|A | 4.15 |
|  |  |  |  |  | SS3 - A\|C\|G\|C\|G\|A | 4.25 |
|  |  |  |  |  | SS4 - Al2O3 Infused A\|C\|G\|C\|G\|A | 4.21 |
|  |  |  |  |  | SS5 - ZrO2 infused A\|C\|G\|C\|G\|A | 4.19 |
|  |  |  |  |  | SS6 - Tio2 infused A\|C\|G\|C\|G\|A | 4.17 |
| [4] | Aluminum with carbon fiber | D 256 | 49.65 | 3 | SS1- A\|C\|A\|C\|A (twill weave) | 9.3 |
|  |  |  |  |  | SS2- C\|A\|C\|A\|C (twill weave) | 5.2 |
|  |  |  |  |  | SS3- C\|C\|C\|C\|C\|C\|C(twill weave) | 1.9 |
| [5] | Aluminum with Kevlar and jute | D 256 | 27.3 | 3 | A\|K\|A\|K\|A\|K\|A | 9.7 |
|  |  |  | 46.7 |  | A\|J\|A\|K\|A\|J\|A | 8.6 |
|  |  |  | 42.9 |  | A\|K\|J\|A\|J\|K\|A | 10.4 |
| [15] | Aluminum with Kevlar and basalt | ISO 179-1 | 25 | 3.25 | A\|BBB\|A\|BBB\|A | 9.162 |
|  |  |  |  | 3.2 | A\|BKB\|A\|BKB\|A | 8.91 |
|  |  |  |  | 3.2 | A\|KBK\|A\|KBK\|A | 8.1 |
|  |  |  |  | 3.17 | A\|KKK\|A\|KKK\|A | 6.75 |
| [16] | Aluminum with basalt | ISO 179-1 | - | 2 | B - FML with 0% nanoclay | 4.78 |
|  |  |  |  |  | B - FML with 1% nanoclay | 4.98 |
|  |  |  |  |  | B - FML with 3% nanoclay | 5.21 |
|  |  |  |  |  | B - FML with 5% nanoclay | 4.87 |

**References**

1. Subramanian, V., Logesh, K., Bright, R. J. & Hariharasakthisudhan, P. Mechanical and impact behaviour of titanium-based fiber metal laminates reinforced with kevlar and jute fibers under various stacking configurations. *Defence Technology* **53**, 19–30 (2025).

2. Wang, Y., Sun, W. & Cao, L. Tensile and flexural mechanical attributes of hybrid carbon/basalt fiber metal laminates under various hybridization and stacking sequences. *Compos. Part A Appl. Sci. Manuf.* **177**, 107942 (2024).

3. Fatima, I., Ejaz, H., Nigar, M., Hussain, R. & A. Khurram, A. Correlation between toughness and metal volume fraction in carbon-glass fiber laminates tested under tensile loading. *Compos. Interfaces* **30**, 1085–1098 (2023).

4. Hynes, N. R. J. *et al.* Effect of stacking sequence of fibre metal laminates with carbon fibre reinforced composites on mechanical attributes: Numerical simulations and experimental validation. *Compos. Sci. Technol.* **221**, 109303 (2022).

5. Hynes, N. R. J. *et al.* Mechanical and microstructural characterization of hybrid fiber metal laminates obtained through sustainable manufacturing. *Archives of Civil and Mechanical Engineering* **22**, 35 (2022).

6. Dahshan, B., El-Habbak, A.-H. M., Adly, M. A. & Shazly, M. Experimental and numerical study on the tensile, three-point-bending, and interlaminar fracture toughness of GLARE. *Journal of Mechanical Science and Technology* **34**, 3273–3281 (2020).

7. Mohammed, I. & Abu Talib, A. R. Mechanical properties of carbon fibre reinforced aluminium laminates using two different layering pattern for aero engine application. *Advances in Materials and Processing Technologies* **5**, 123–131 (2019).

8. Gao, S., Hou, W., Xing, J. & Sang, L. Numerical and Experimental Investigation of Flexural Properties and Damage Behavior of CFRTP/Al Laminates with Different Stacking Sequence. *Applied Sciences* **13**, 1667 (2023).

9. Hosseini Abbandanak, S. N. *et al.* Effect of graphene on the interfacial and mechanical properties of hybrid glass/Kevlar fiber metal laminates. *Journal of Industrial Textiles* **51**, 2576S-2593S (2022).

10. Gupta, R. K., Mahato, A. & Bhattacharya, A. Strength and failure behavior of carbon fiber reinforced aluminum laminates under flexural loading. *Mechanics of Advanced Materials and Structures* **29**, 662–676 (2022).

11. Bellini, C., Di Cocco, V., Iacoviello, F. & Sorrentino, L. Performance evaluation of CFRP/Al fibre metal laminates with different structural characteristics. *Compos. Struct.* **225**, 111117 (2019).

12. Li, H., Xu, Y., Hua, X., Liu, C. & Tao, J. Bending failure mechanism and flexural properties of GLARE laminates with different stacking sequences. *Compos. Struct.* **187**, 354–363 (2018).

13. Azghan, M. A. & Eslami-Farsani, R. The effects of stacking sequence and thermal cycling on the flexural properties of laminate composites of aluminium-epoxy/basalt-glass fibres. *Mater. Res. Express* **5**, 025302 (2018).

14. Kali, N., Korla, R. & Korla, S. Impact Behaviour of Nano-Hybrid (Carbon/Glass) Fibre Metal Laminates: An Experimental Study. *Arab. J. Sci. Eng.* **48**, 3881–3891 (2023).

15. Arpatappeh, F. A., Azghan, M. A. & Eslami-Farsani, R. The effect of stacking sequence of basalt and Kevlar fibers on the Charpy impact behavior of hybrid composites and fiber metal laminates. *Proc. Inst. Mech. Eng. C J. Mech. Eng. Sci.* **234**, 3270–3279 (2020).

16. Bahari-Sambran, F., Meuchelboeck, J., Kazemi-Khasragh, E., Eslami-Farsani, R. & Arbab Chirani, S. The effect of surface modified nanoclay on the interfacial and mechanical properties of basalt fiber metal laminates. *Thin-Walled Structures* **144**, 106343 (2019).
